# Expression profiling of inhibitory immune checkpoints in colorectal cancer stem cells and their association with tumor immunity and immunotherapy biomarkers

**DOI:** 10.3389/fimmu.2026.1760555

**Published:** 2026-04-08

**Authors:** Ola J. Hussein, Muhammad Ammar Zahid, Hanan H. Abunada, Abdelali Agouni, Cristina Maccalli, Hesham M. Korashy

**Affiliations:** 1 Department of Pharmaceutical Sciences, College of Pharmacy, QU Health, Qatar University, Doha, Qatar; 2 Biomedical Research Center, QU Health, Qatar University, Doha, Qatar; 3 Research Branch, Sidra Medicine, Doha, Qatar; 4 Unit of Biotherapies, Scientific Institute for Research and Health Care (IRCCS) Azienda Ospedaliera Metropolitana Plesso Policlinico San Martino, Genova, Italy

**Keywords:** cancer stem cells, colorectal cancer, immune checkpoints, immunotherapy, mRNAsi, stemness

## Abstract

**Background:**

Colorectal cancer stem cells (CSCs) represent a rare subpopulation within tumors endowed with self-renewal capabilities and are recognized as key drivers of tumor initiation, metastasis, and therapeutic resistance. These cells also display immunomodulatory properties that enable them to evade immune surveillance. However, the mechanisms underlying their immune evasion, including the role of immune checkpoints (ICPs), remain poorly understood. Therefore, this study aimed to characterize the inhibitory ICP expression landscape in colorectal CSC-enriched models and to evaluate its association with tumor microenvironment and biomarkers related to immune checkpoint inhibitor (ICI) response.

**Methods:**

CSC-enriched spheroids, cancer stem-like cells (CSLCs), were generated from four colorectal cancer cell lines (HCT-116, HT-29, SW480, SW620). Differential expression of stemness markers and inhibitory ICPs between spheroid cultures and bulk cancer cells were assessed by real-time PCR, Western blotting, Immunofluorescence, and flow cytometry. Additionally, RNA-seq and clinical data from colorectal adenocarcinoma patients in The Cancer Genome Atlas (TCGA) were retrieved and stratified using the mRNA expression-based stemness index (mRNAsi), a stemness score derived using the one-class logistic regression machine learning algorithm. Correlations between cancer stemness and the tumor immune microenvironment, as well as ICIs-related biomarkers, including ICP expression levels, tumor mutational burden (TMB), and microsatellite instability (MSI), were subsequently analyzed.

**Results:**

Spheroid cultures exhibited a significant elevation in the expression of stemness markers (e.g., ALDH, NANOG, and SOX9), confirming the successful enrichment of CSC subpopulations. This upregulation was accompanied by increased expression of multiple inhibitory ICPs (e.g., PD-L1, B7-H3, and CD47) compared with their parental adherent cells (cancer), suggesting a potential role for these ICPs in mediating CSC characteristics. Consistently, patients with high stemness scores displayed reduced immune cell infiltration, increased TMB, higher MSI prevalence, and elevated expression of multiple ICPs, after adjusting for tumor purity, indicating an association between the tumor stemness and factors predictive of ICI responsiveness.

**Conclusion:**

The unique immunological profile of colorectal CSLCs identified in this study highlights the role of ICPs in CSC-mediated immune evasion and underscores the potential of CSCs both as targets for checkpoint blockade-based immunotherapies and as biomarkers of response.

## 1 Introduction

Colorectal cancer (CRC) is the third most frequently diagnosed solid tumor and the second leading cause of cancer-related mortality worldwide (1). Despite the decline in CRC mortality rates since 1990, approximately 25% of CRC patients still present with stage IV disease at the time of diagnosis, and an additional 25-50% of treated patients experience recurrence and progress to metastatic disease (2). Unlike localized CRC, patients presenting with metastatic CRC (mCRC) have a poor prognosis with a dramatically reduced median 5-year survival rate of about 12.5% (3, 4). Multiple therapeutic options have been developed and employed for the management of mCRC, aiming to reduce its morbidity and mortality, yet the disease remains essentially incurable.

One recent theory of cancer recurrence and therapy resistance is the presence of a stem-like cell population within tumors, known as cancer stem cells (CSCs), cancer stem-like cells (CSLCs) or cancer-initiating cells (CICs). CSCs represent a minor subpopulation of tumor cells endowed with stemness properties, including self-renewal capacity, unlimited proliferation, and multipotency (5, 6). Clinically, enrichment of colorectal CSCs, as evidenced by upregulation of stemness markers, has been repeatedly associated with disease progression and a poorer prognosis (7–9). CSCs are thought to be responsible for tumor initiation, progression, metastasis, recurrence, and resistance to conventional therapies (10). The ability of these cells to cycle between proliferation and quiescence, increased expression of anti-apoptotic and drug efflux proteins, and upregulation of DNA repair molecules are among the principal molecular alterations that drive their resistance to therapy (11–14). Nevertheless, other key mechanisms underlying this phenomenon still need to be dissected and fully understood.

Another key hallmark of CSCs that has attracted much attention over the past few years is their ability to evade immune surveillance and influence the response to immunotherapies (15–17). For instance, Volonté et al. reported that colorectal CICs exhibited reduced immunogenicity and elicited weaker T-cell responses when co-cultured with PBMCs compared with their non-CIC counterparts (18). Few immunomodulatory properties associated with CSCs have been reported, including the secretion of immunosuppressive cytokines/factors, such as growth differentiation factor-15 (GDF-15), interleukin-10 (IL-10), interleukin-13 (IL-13), and transforming growth factor-β (TGF-β) (18–20). These factors drive the differentiation of immune cells towards suppressive subtypes such as regulatory T cells (Tregs) and myeloid-derived suppressor cells (MDSCs) (19–21). Moreover, some studies have reported that CSCs express suboptimal levels of human leukocyte antigen (HLA) molecules, which render these cells invisible to immune cells, potentially leading to the T cells’ inability to recognize and kill CSCs (22). On the other hand, Agudo et al. showed that immune evasion in intestinal epithelial stem cells is state-dependent, with quiescent—but not cycling—stem cells downregulating antigen-processing and presentation machinery (23). Nevertheless, further studies are needed to fully dissect immune evasive mechanisms in CSCs.

Advances in immunotherapy over the past few years have changed the paradigm of cancer therapy (2). Notably, the clinical development of monoclonal antibodies antagonizing the signaling of inhibitory immune checkpoints (ICPs), such as programmed cell death (PD-1)/PD-L1 or cytotoxic T lymphocyte antigen-4 (CTLA-4) signaling, represents a breakthrough in immunotherapy (24–26). ICP inhibitors (ICIs) have shown unprecedented success in achieving long-term durable responses in aggressive solid tumors, including a subset of CRC patients with high microsatellite instability (MSI), which received FDA approval in 2017 (27, 28). However, a significant proportion of cancer patients do not respond to these therapies due to primary or acquired resistance (29, 30). Therefore, identifying biomarkers predictive of clinical response and developing strategies to overcome resistance are critical unmet needs. Additionally, whether the available ICIs could effectively target CSCs is not well established. While some studies reported upregulation of PD-L1 expression in CSCs of head and neck squamous cell carcinoma (31), breast (32), and colon cancers (33), others showed decreased or no significant difference in PD-L1 expression between CSCs and non-CSCs (34, 35). Evidence indicate that CSCs may utilize alternative ICPs, such as B7-H3, B7-H4, CD200, CEACAMs, to evade immune surveillance and potentially mediate resistance to ICIs (36). The engagement of these inhibitory molecules with their receptors on T cells and other immune cells may contribute to inefficient immune responses and promote CSC survival.

To date, the full array of ICPs expressed by CSCs and their relation to ICI response remains undefined (37, 38). Studies in CSCs have been heavily skewed toward the PD-1/PD-L1 axis, with limited data on the expression and regulation of other ICPs (e.g., PD-L2, CD200, B7-H3, CD155, LAG3, CD47, CD70, CD80, CD86, B7-H4, HVEM) (36). A significant proportion of CRC patients do not respond to or develop resistance to anti-PD-1/PDL-1 therapies, highlighting the importance of investigating the role of other ICPs that can mediate immune evasion, especially in the CSC subpopulation, which is endowed with highly tumorigenic properties. Accordingly, in this study we systematically profiled a panel of inhibitory ICPs in colorectal CSC-enriched spheroid cultures and integrated these findings with TCGA-COAD stemness analyses, revealing upregulation of multiple ICPs in CSC-enriched cells (notably PD-L1, B7-H3, and CD47) and linking high tumor stemness with alterations in the tumor immune microenvironment and biomarkers relevant to response to ICIs.

## 2 Materials and methods

### 2.1 Cell lines and culture conditions

Human colorectal cancer cell lines (HCT-116, HT-29, SW480, SW620) were obtained from the American Type Culture Collection (ATCC). Cells were cultured as adherent monolayers in high-glucose (4.5 g/L) Dulbecco’s Modified Eagle’s Medium (DMEM) with GlutaMAX (Gibco, cat. 31966-047) supplemented with 10% heat-inactivated fetal bovine serum (FBS) and 1% Antibiotic–Antimycotic (Gibco, cat. 15240-062) and maintained at 37 °C in a humidified incubator with 5% CO_2_. All cell lines were tested and confirmed to be mycoplasma-free.

### 2.2 Spheroid culture

To generate CSC-enriched spheroids (CSLCs), CRC cells were harvested and seeded at a density of 50, 000 cells/mL in ultra-low-attachment T-75 flasks (Nunclon Sphera, Thermo Fisher Scientific) and cultured in serum-free StemFlex medium (Gibco, A3349401) supplemented with 1× Antibiotic-Antimycotic in a humidified 5% CO2 incubator at 37 °C. For sequential passages, established spheroids were collected by gravitational sedimentation for 10 min, dissociated enzymatically into single cells using 1× TrypLE Express (Gibco, 12605-028) and mechanically by gentle pipetting. The resulting single cells were then reseeded at the same density and under the same culture conditions described above (39, 40).

### 2.3 RNA isolation and reverse transcription-quantitative polymerase chain reaction

Total RNA was extracted from cells using PureLink™ RNA Mini Kit (Invitrogen, cat. 12183025) according to the manufacturer’s instructions. The purity and concentration of RNA were determined by NanoDrop™ 8000 Spectrophotometer (Thermo Scientific). Next, 1 μg of total RNA was reverse-transcribed using the High-Capacity cDNA Reverse Transcription Kit (Applied Biosystems, cat. 4374966) as per the manufacturer’s instructions. qPCR reactions were performed on QuantStudio™ 12K Flex Real-Time PCR System using PowerUp™ SYBR™ Green Master Mix (Applied Biosystems, cat. A25742) (41). Relative expression of target genes was calculated by the comparative ΔΔCt method using GAPDH as the housekeeping gene. The PCR primer sequences used are listed in **Supplementary Table 1**.

### 2.4 Western blotting analysis

Colon CSLCs and bulk tumor cells were washed with ice-cold PBS and total proteins were extracted using radioimmunoprecipitation assay (RIPA) lysis buffer containing 1× Halt™ Protease Inhibitor Cocktail (Thermo Scientific, cat. 78429). Protein concentrations were quantified using the Pierce™ Rapid Gold BCA Protein Assay Kit (Thermo Scientific, cat. A53225) according to the manufacturer’s instructions. Equal amounts of protein (30 μg) were separated by 10% sodium dodecyl sulfate-polyacrylamide gel electrophoresis (SDS-PAGE) and then transferred to a 0.45-µm polyvinylidene fluoride (PVDF) membrane. Subsequently, membranes were blocked with 5% bovine serum albumin (BSA) (Fisher Scientific, cat. BP9702-100) in TBST for 1 hour at room temperature and incubated with primary antibodies (**Supplementary Table 2**) diluted in 5% BSA in TBST at 4 °C overnight. Membranes were then washed before being incubated with HRP-conjugated secondary antibodies for 1.5 hours at room temperature. Finally, immunoblots were developed by SuperSignal™ West Pico PLUS Chemiluminescent Substrate (Thermo Scientific, cat. 34580), and blots were imaged on a ChemiDoc Imaging System (Bio-Rad). Densitometry analysis was carried out using ImageJ.

### 2.5 ALDEFLUOR assay

The ALDH activity was detected with the ALDEFLUOR™ kit (STEMCELL Technologies, cat. 01700) following the manufacturer’s instructions. Briefly, cancer cells and their derived CSLCs were harvested, and spheroids were dissociated into single cells with TrypLE™ Express. Cells were then washed in phosphate-buffered saline (PBS). Immediately after resuspension, 500 µL of the cell suspension was transferred to a tube containing diethylaminobenzaldehyde (DEAB), a broad inhibitor of ALDH, at a final concentration of 15 μM, which served as a reference control for background fluorescence. Test (−DEAB) and control (+DEAB) samples containing ALDEFLUOR™ reagent were incubated for 30 min at 37 °C. Subsequently, cells were centrifuged, resuspended in 500 µL cold assay buffer, and stored on ice until analyzed. The brightly fluorescent ALDH-expressing (ALDH^+^) cells were detected on a BD FACSAria™ III (BD Biosciences), and doublets were excluded by forward- and side-scatter gating. Specific ALDH activity was determined based on the difference between the presence/absence of the ALDEFLUOR inhibitor DEAB. Data were analyzed using FlowJo™ software. Each experiment was repeated at least three times.

### 2.6 Flow cytometry analysis

For detection of surface markers, cells were washed twice with FACS buffer (ice-cold PBS containing 2% FBS) and incubated with antibodies diluted in FACS buffer for 30 min at 4 °C in the dark. When unconjugated primary antibodies were used, cells were incubated with primary antibodies and subsequently stained with appropriate Alexa Fluor 647-conjugated goat secondary antibodies for 30 min at 4 °C. The cells were then washed twice and stained with 1 µg/mL DAPI for 2 min immediately prior to acquisition to exclude non-viable cells. Samples were acquired on BD FACSAria™ III (BD Biosciences) and analyzed with FlowJo™ v10.10.0. Gating was performed using matched isotype controls. All antibodies are listed in **Supplementary Table 3**.

### 2.7 Immunofluorescence

CSLCs and parental adherent cells (cancer) were grown on sterile coverslips, rinsed twice with PBS and fixed in 4% paraformaldehyde (PFA) in PBS for 15 min at room temperature. Cells were permeabilized with 0.2% Triton X-100 in PBS for 10 min, then blocked with 10% FBS in PBS for 1 h at room temperature in a humidified chamber. After removing the blocking solution, cells were incubated with the following primary antibodies diluted in blocking buffer: ALDH1A1 (Invitrogen, cat. MA5-29023; 1:100), SOX9 (SantaCruz, cat. sc-166505; 1:50), NANOG (SantaCruz, cat. sc-293121; 1:50), or Cytokeratin Pan Type I (Invitrogen, cat. MA5-13144; 1:100) at 4 °C overnight (**Supplementary Table 4**). The next day, the coverslips were washed three times with PBS and incubated with Alexa Fluor 594-conjugated goat anti-mouse IgG secondary antibody (Invitrogen, cat. A11005; 1:500) for 2 h at room temperature in the dark. Nuclei were counterstained with DAPI (1 µg/mL, 2 min), followed by two PBS rinses, and coverslips were mounted with ProLong™ Glass Antifade Mountant (Invitrogen, cat. P36984). Slides were protected from light and were examined and imaged on an EVOS™ M5000 Imaging System (Invitrogen) using identical exposure settings across groups.

### 2.8 siRNA knockdown

Specific siRNAs targeting B7-H3 (Santa Cruz Biotechnology, sc-45444) or CD155 (sc-61903), as well as a control siRNA (sc-37007), were purchased from Santa Cruz Biotechnology. Cancer cells were seeded at 2 × 10^5^ cells/well in 6-well plates in complete culture medium. After 24 h, cells were transfected with the indicated siRNAs using Lipofectamine™ RNAiMAX (Invitrogen, cat. 13778-030) in Opti-MEM™ (Gibco, cat. 31985062) according to the manufacturer’s instructions. Following 8 h incubation at 37 °C, 1 mL of 2× complete growth medium was added directly to each well without removing the transfection mixture. Cells were harvested and used for the indicated assays 48 h post-transfection.

### 2.9 Kaplan–Meier plotter

To analyze the prognostic value of inhibitory ICPs in CRC patients, we used the Kaplan–Meier Plotter (https://kmplot.com/analysis/, accessed on September 8, 2025). Three prognostic indices, overall survival (OS), recurrence-free survival (RFS), and post-progression survival (PPS), were included. CRC patients were divided into two groups (low and high) based on the median expression level, and the survival was assessed using Kaplan–Meier survival plots. Log-rank P value, hazard ratios (HRs), and 95% confidence intervals (CIs) were automatically generated. Log-rank P value < 0.05 was considered statistically significant.

### 2.10 TNMplot

TNM_plot (https://tnmplot.com/analysis/, accessed on September 5, 2025) is an online platform that enables comparison of gene expression in normal, tumor, and metastatic tissues. The database includes RNA-seq data from The Cancer Genome Atlas (TCGA), The Genotype-Tissue Expression Project (GTEx), and Therapeutically Applicable Research to Generate Effective Treatments (TARGET). We used TN-plot tool to compare the expression of ICPs between tumor tissues from CRC patients and normal colon tissue samples from non-cancerous patients based on RNA-seq data. The differences were assessed using the Mann–Whitney U test. For metastatic versus primary tumor comparisons, GeneChip-based datasets from TNMplot were used, as metastatic RNA-seq data for colorectal cancer were not available within the platform. Differences among normal, primary tumor, and metastatic tissues were assessed using the Kruskal–Wallis test, followed by Dunn’s *post hoc* test for multiple comparisons, as implemented by TNMplot.

Additionally, the platform was utilized to examine the Gene-vs-Gene correlation between selected stemness and inhibitory ICPs in CRC patients. The correlation was evaluated by using Spearman’s rank correlation, and P values < 0.05 were considered statistically significant.

### 2.11 Computation of stemness score

RNA-seq data and corresponding clinical characteristics of 458 colorectal adenocarcinoma (COAD) tumor samples were downloaded from the TCGA database (https://portal.gdc.cancer.gov/), current as of September 17, 2025. Stemness indices (mRNAsi) were calculated based on a One-Class Logistic Regression (OCLR) machine learning algorithm developed by Malta et al. (35). mRNAsi values ranged from 0 to 1, with higher scores indicating greater tumor dedifferentiation and enhanced stem cell activity.

### 2.12 Differential expression analysis and functional annotation

The COAD tumor samples were divided into two groups, ‘High_stemness’ and ‘Low_stemness’, by splitting the cohort at the median of the calculated mRNA stemness index (mRNAsi). Differential gene expression (DGE) analysis between these two groups was performed using the limma package. A linear model was fitted to the expression data for each gene, and contrasts were defined to compare the High_stemness and Low_stemness groups directly. Empirical Bayes moderation was applied to the standard errors to generate more stable and reliable statistical inference. A gene was defined as significantly differentially expressed if it exhibited an absolute log2 fold change (logFC) greater than 1 and a P value, adjusted for multiple testing using the Benjamini-Hochberg method, of less than 0.05. Results were visualized using a volcano plot generated with the ggplot2 and ggrepel packages in R (v 4.4.2). To understand the biological significance of the differentially expressed genes, functional enrichment analysis was performed separately on the upregulated and downregulated gene sets. The clusterProfiler and ReactomePA R packages were utilized to conduct Gene Ontology (GO), Kyoto Encyclopedia of Genes and Genomes (KEGG), and Reactome pathway analyses. For each analysis, a hypergeometric test was used to assess the over-representation of our gene lists within established pathways. The resulting P values were adjusted for multiple comparisons using the Benjamini-Hochberg method, and terms with a q-value < 0.05 were deemed significantly enriched (42, 43).

### 2.13 Analysis of tumor purity and immune infiltration

Tumor purity and the presence of infiltrating immune and stromal cells in the tumor microenvironment were estimated using the tidyestimate (v 1.1.1) R package, which is a tidy implementation of the “ESTIMATE” R package (44). The algorithm generates three scores: an Immune Score reflecting the level of immune cell infiltration, a Stromal Score representing stromal cell infiltration, and an ESTIMATE Score, a combined immune-stromal metric that infers tumor purity. To explore the differences in immune cell phenotypes between the high- and low-stemness groups, immune cell infiltration in each COAD sample was estimated using the “immunedeconv” R package, which includes several established deconvolution algorithms (xCELL, TIMER, MCPCOUNTER, quanTIseq, EPIC, and CIBERSORT) (45).

### 2.14 Tumor mutation burden

Precomputed TMB scores based on nucleotide variation data were used from the tmb_tcga dataset using the UCSCXenaShiny package in R (46).

### 2.15 Prediction of response to immune checkpoint inhibitors

The Tumor Immune Dysfunction and Exclusion (TIDE) webserver (http://tide.dfci.harvard.edu/) was used to predict the response of COAD patients to ICIs, with lower TIDE scores indicating a higher likelihood of response to immunotherapy (47, 48).

### 2.16 Statistical analysis

Statistical analyses were performed using GraphPad Prism version 10 (San Diego, CA, USA). Differences between CSLCs and parental adherent cells (cancer) were determined by an unpaired, two-tailed Student’s t-test. The data were presented as mean ± SEM from at least three independent experiments (n ≥ 3). Results were considered statistically significant when P values were < 0.05. Statistical significance is expressed as *, P < 0.05, **, P < 0.01 and ***, P < 0.001. All statistical computations and data visualizations related to mRNAsi were conducted using the R programming environment (v 4.4.2). Data manipulation and preparation were performed using the Tidyverse suite of packages, primarily dplyr. For visualization, the ggplot2 package, based on the Grammar of Graphics, was used to generate publication-quality figures. Standard statistical comparisons, such as Student’s t-tests, Wilcoxon rank-sum tests, and Pearson’s correlations, were performed using functions from the base stats package.

## 3 Results

### 3.1 Enrichment of CSLCs by spheroid culture of established CRC cell lines

**Figure 1:**
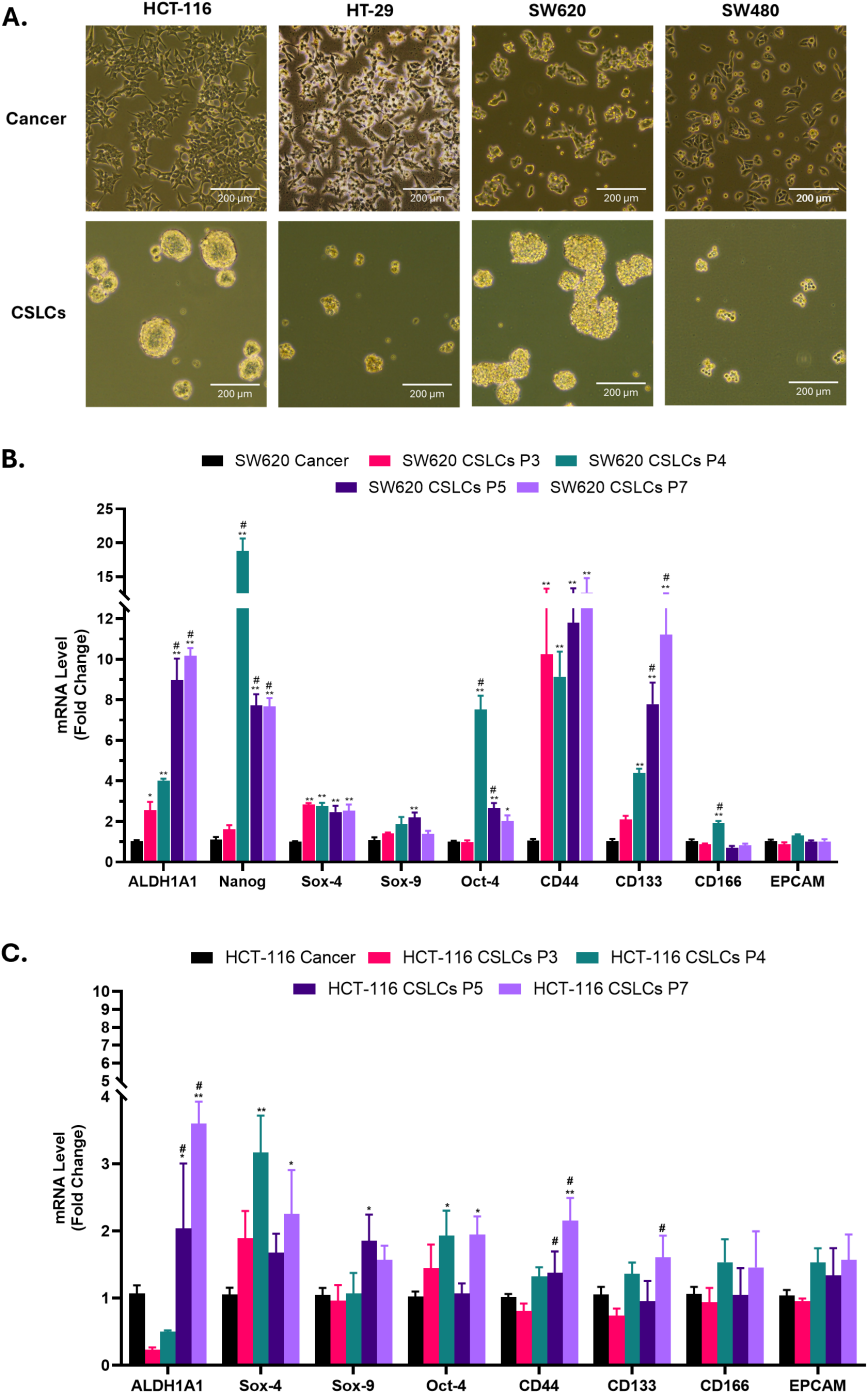
Enrichment of colorectal cancer stem-like cells (CSLCs) by spheroid culture. **(A)** Morphological examination of colorectal cancer cell lines and their derived CSLCs. Phase-contrast images of HCT-116, HT-29, SW620 and SW480 cells cultured under adherent monolayer conditions (Cancer) or as spheroid CSLC-enriched cultures under low-attachment stem-cell conditions. Cells were maintained either in standard culture medium (DMEM supplemented with 10% FBS) or in Stem Flex medium (see Methods). Images were taken using a 10x objective (n = 3). The scale bar represents 200 μm. **(B, C)** RT-qPCR analysis of stemness-associated genes across different passages of SW620 **(B)** and HCT-116 **(C)** spheroid cultures (CSLCs) compared with their respective parental adherent (cancer) cells. mRNA levels were normalized to the housekeeping gene GAPDH and are shown as fold change relative to cancer cells. Statistical significance was determined by one-way ANOVA followed by Tukey’s post hoc test. *P < 0.05 and **P < 0.01 versus parental adherent (cancer) cells; #P < 0.05 versus earlier passage (P3); n ≥ 3.

In this study, we aimed to enrich CSCs from four colorectal cancer cell lines, HCT-116, HT-29, SW620 and SW480. Given the lack of a single, definitive, and universally accepted CSC marker and the heterogeneity within CSC subpopulations, we utilized 3D spheroid cultures to enrich cancer stem or stem-like cells, denominated here as CSLCs. To generate tumor spheres, cells were enzymatically dissociated and seeded into ultra-low-attachment flasks in serum-free stem cell medium. Under these conditions, a subset of cells died due to serum starvation, whereas the surviving sphere-forming cells (CSLCs) persisted and subsequently proliferated in suspension, giving rise to multicellular spheroids. Both HCT-116 and HT-29 produced round, compact spheroids, while SW620- and SW480-derived spheroids were more loosely aggregated (**Figure 1A**). Unlike spheroid cultures from other types of tumors that are often maintained for ~2–3 weeks, we noticed that CRC spheroids began to lose integrity and partially dissociated by days 5–7 of culture under serum-free, low-attachment conditions.

Given that serial passaging has been reported to enhance CSC traits and that the ability of spheroids to regrow upon passaging reflects their self-renewal capacity (49), serial passaging has been performed. Spheres were dissociated into single cells and replated three times per week, yielding secondary spheroids. Additionally, we used gravitational sedimentation before each passage to collect spheroids, thereby separating them from single non-sphere-forming cells, and further enriching CSCs (50). This approach produced sustainable spheroids from SW620 and HCT-116 cell lines under stem-selective conditions. By contrast, the sphere-forming ability of HT-29 and SW480 declined markedly over serial passages, consistent with previous observation by Gheytanchi et al. (51). Therefore, HCT-116 and SW620 cell lines were selected for subsequent experiments.

**Figure 2:**
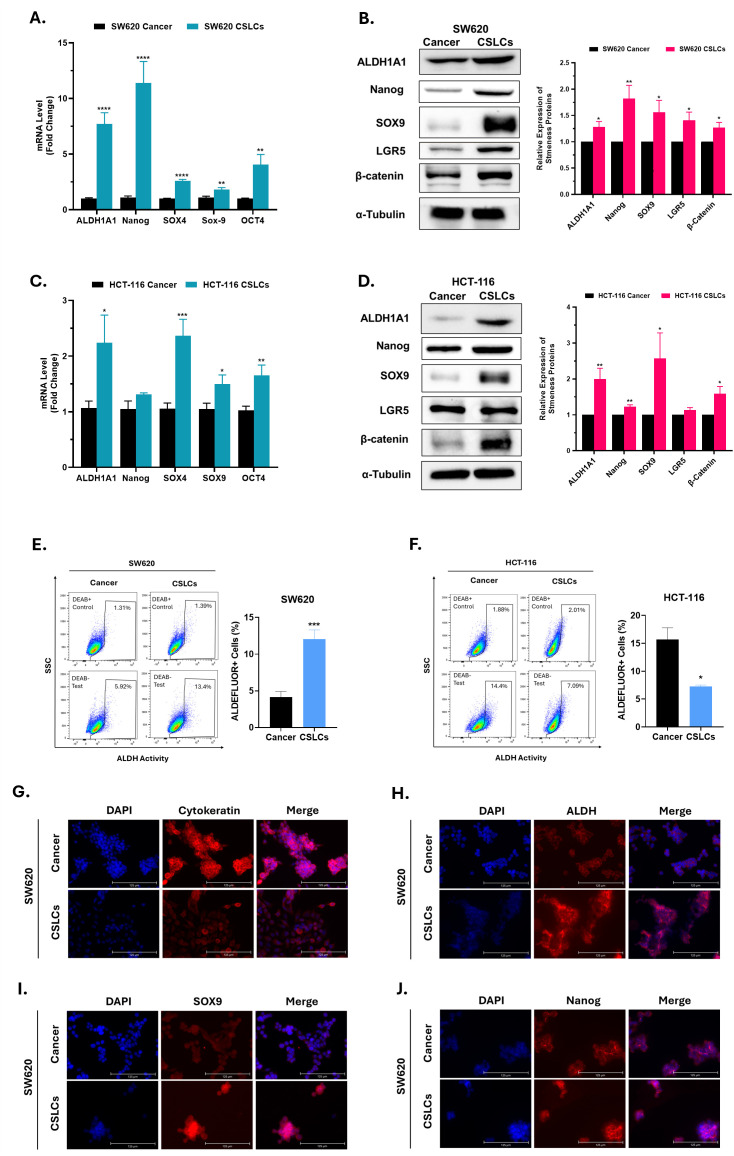
Expression of stemness regulators in colorectal CSLCs. This figure illustrates differential expression of stemness regulators in spheroid cultures (CSLCs) compared with parental adherent cells (cancer). **(A, C)** RT-qPCR analysis of stemness-associated genes in SW620 and HCT-116 cell lines, respectively. mRNA levels were normalized to the housekeeping gene GAPDH and are shown as fold change relative to cancer cells. **(B, D)** Immunoblot analysis of stemness regulators in SW620 and HCT-116, respectively, using α-Tubulin as a housekeeping protein. The right panels show densitometric quantification of protein levels in CSLCs relative to cancer cells. **(E, F)** ALDEFLUOR™ assay of ALDH activity in SW620 and HCT-116, respectively. Left: representative flow cytometry plots. DEAB, a specific ALDH inhibitor, was used as a control to establish a baseline fluorescence and define the ALDEFLUOR™-positive gate. Right: quantification of the ALDEFLUOR+ cells. **(G-J)** Representative immunofluorescence images showing the expression of cytokeratin, ALDH1A1, SOX9, and Nanog, respectively, in SW620 cancer and CSLCs—scale bar: 125 μm. Data represent mean ± SEM. *P < 0.05, **P < 0.01, ***P < 0.001, and ****P < 0.0001 by Student’s t-test; n ≥ 3.

To confirm that our spheroid culture enriched the CSC populations, we first evaluated the expression of a wide range of stemness regulators in both sphere cultures (CSLCs) and their parental adherent tumor cells using several approaches. Although stemness-associated genes started increasing from early passages, we observed a tendency for a progressive increase in their expression with serial passaging of spheroid cultures (**Figures 1B, C** and **S1**), suggesting further enrichment of CSCs. Accordingly, spheroids were serially passaged three times prior to use in subsequent experiments. Notably, RT-qPCR analysis revealed significant upregulation of the majority of examined stem cell regulators in both HCT-116 and SW620 CSLCs compared with adherent cancer cells. In particular, the mRNA expression of ALDH1A1, OCT4, SOX4 and SOX9 increased significantly in CSLCs of both cell lines, which was more pronounced in SW620 CSLCs than in HCT-116 CSLCs (**Figures 2A, C**). Similar results were observed at the protein level. Western blot analysis (**Figures 2B, D**) showed that spheroid cultures exhibited significant elevation in multiple stemness regulators (i.e., ALDH1A1, Nanog, SOX9, and β-catenin) in both cell lines. On the other hand, LGR5 expression was significantly increased only in SW620 CSLCs.

Next, to further confirm CSC enrichment, ALDH activity was assessed by flow cytometry using the ALDEFLUOR™ assay, with DEAB, an ALDH inhibitor, as a negative control. In SW620, ALDH activity was significantly higher in CSLCs than in cancer cells (**Figure 2E**). Conversely, HCT-116 CSLCs showed lower ALDH activity than their parental counterparts (7.27 ± 0.35% vs. 15.7 ± 2.96%; *p* = 0.0162) (**Figure 2F**).

We next compared the expression of key stemness regulators and a selected differentiation marker by immunofluorescence. Each cell line, parental adherent cells (cancer) or spheroids (CSLCs), was imaged for its expression of cytokeratin, ALDH1A1, SOX9, and Nanog. All assessed markers were detectable in both CSLCs and parental cancer cells (**Figures 2G–J**); however, CSLC cultures generally displayed higher intensities of stemness markers (ALDH1A1, SOX9, and Nanog) and lower expression of the differentiation marker (cytokeratin), particularly in SW620.

### 3.2 Expression of stemness markers in spheroid-enriched colorectal CSCs

**Figure 3:**
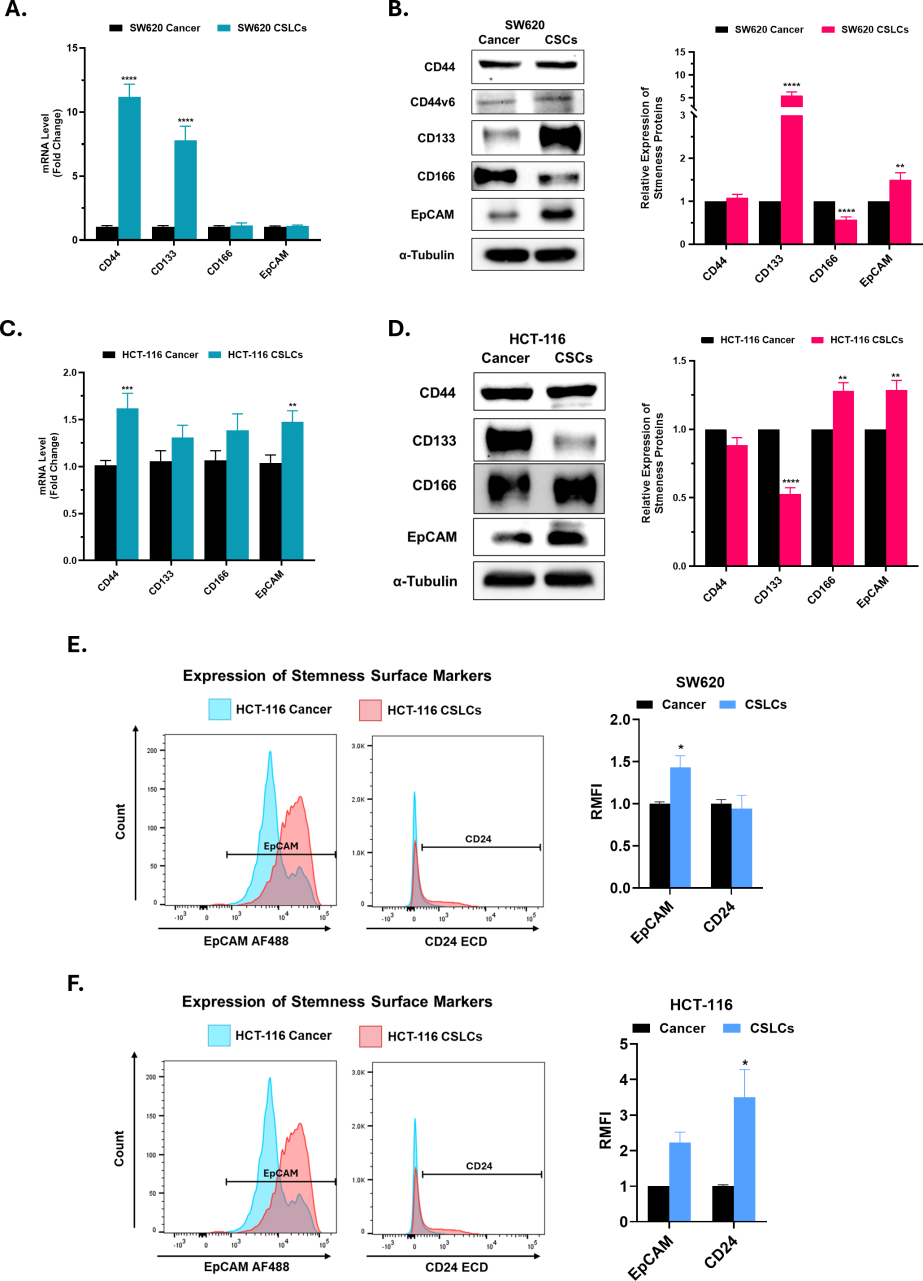
Expression of stemness markers in spheroid-enriched colorectal CSLCs. This figure illustrates the differential expression of stemness-associated markers in spheroid cultures (CSLCs) compared with parental adherent cells (cancer). **(A, C)** RT-qPCR analysis of stemness-associated markers in SW620 and HCT-116 cell lines, respectively. mRNA levels were normalized to the housekeeping gene GAPDH and are shown as fold change relative to cancer cells. **(B, D)** Immunoblot analysis of stemness markers in SW620 and HCT-116, respectively, using α-Tubulin as a housekeeping protein. The right panels show densitometric quantification of protein levels in CSLCs relative to cancer cells. **(E, F)** Representative flow cytometry histograms showing surface expression of stemness-associated markers in CSLCs (red) and cancer cells (blue) for SW620 and HCT-116, respectively. The right panels show the relative mean fluorescence intensity (RMFI) for each marker in CSLCs, normalized to cancer cells. Data represent mean ± SEM. *P < 0.05, **P < 0.01, ***P < 0.001, and ****P < 0.0001 by Student’s t-test; n ≥ 3.

Second, in addition to stemness regulators, we examined the expression of several putative CSC surface markers at both the mRNA and protein levels (**Figures 3A–D**). SW620-derived spheroids showed a significant increase in CD133 and EpCAM, whereas HCT-116 spheroids upregulated EpCAM and CD166. Conversely, CD166 was significantly downregulated in SW620, and CD133 was downregulated in HCT-116 spheroids. Although CD44 was significantly upregulated at the mRNA level in CSLCs of both cell lines, no corresponding increase was detected at the protein level. Surface EpCAM expression, assessed by flow cytometry, was positive in ~100% of cells in both cell lines, with higher signal intensity in CSLCs than in parental cancer cells, consistent with Western blot findings (**Figures 3E, F** and **Supplementary Figure S2**). CD24 surface expression was upregulated only in HCT-116 CSLCs, not SW620 CSLCs (**Figures 3E, F** and **Supplementary Figure S2**). Collectively, these findings demonstrate successful CSC enrichment in SW620 and HCT-116 and underscore the heterogeneity among CSC populations.

### 3.3 CSLCs differentially express inhibitory immune checkpoints

**Figure 4:**
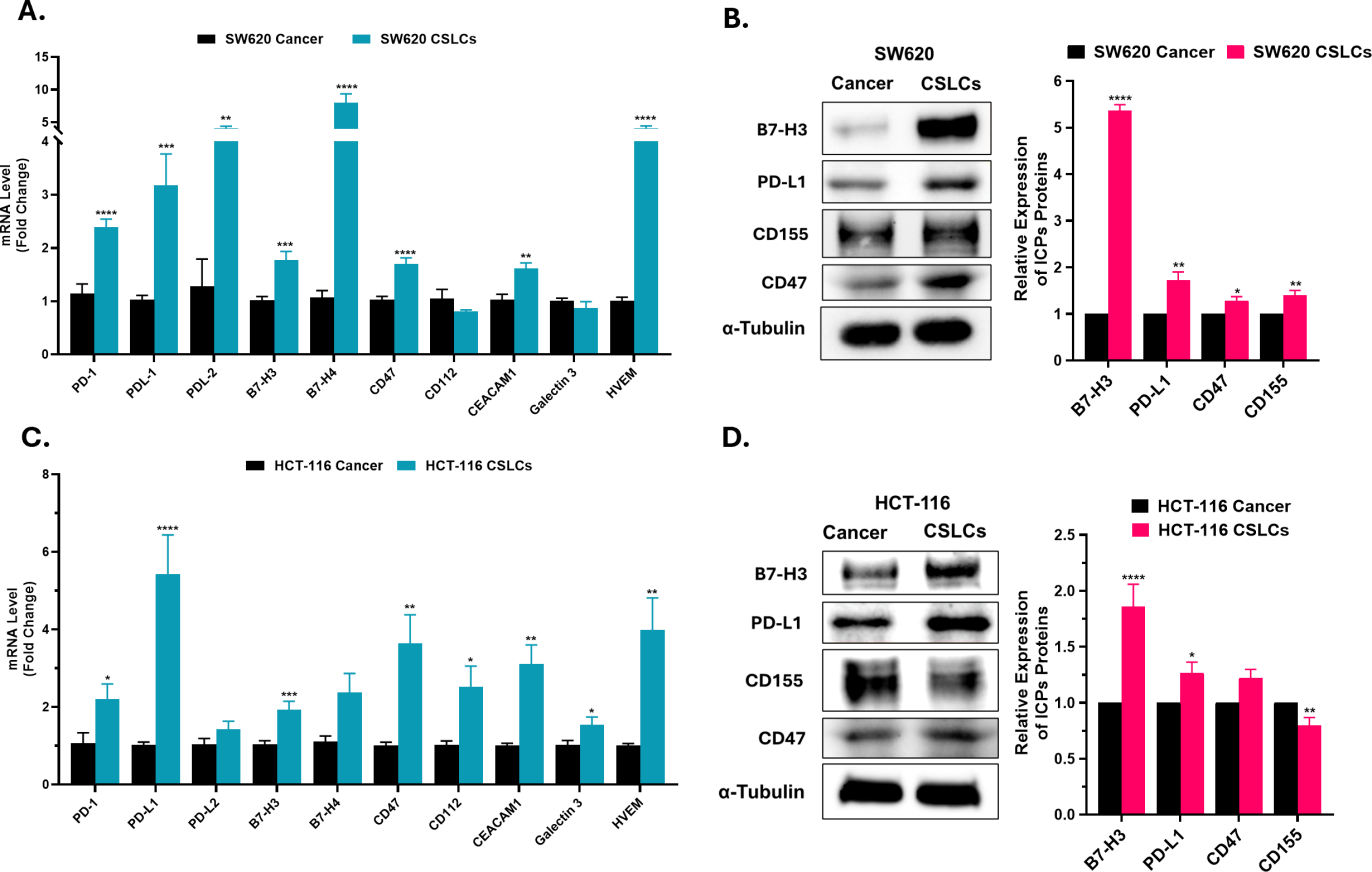
Inhibitory immune checkpoints (ICPs) expression in spheroid-enriched CSLCs. This figure illustrates the differential expression of various ICPs between spheroids (CSLCs) and parental adherent cells (cancer). **(A, C)** RT-qPCR analysis of ICP genes in SW620 and HCT-116 cell lines, respectively. mRNA levels were normalized to the housekeeping gene GAPDH and are shown as fold change relative to cancer cells. **(B, D)** Immunoblot analysis of ICP molecules in SW620 and HCT-116, respectively, using α-Tubulin as a housekeeping protein. The right panels show densitometric quantification of protein levels in CSLCs relative to cancer cells. Data represent mean ± SEM. *P < 0.05, **P < 0.01, ***P < 0.001, and ****P < 0.0001 by Student’s t-test; n ≥ 3.

To determine inhibitory immune checkpoint expression in CSLCs, we quantified the mRNA and protein levels of several ICPs. RT-qPCR revealed that CSLCs upregulate multiple ICPs relative to parental cells, including PD-1 (*PDCD1*), PD-L1 (*CD274*), B7-H3 (*CD276*), *CD47*, *CEACAM1*, and HVEM (*TNFRSF14*) in both cell lines compared with parental adherent cells (cancer). On the other hand, PD-L2 (*PDCD1LG2*) and B7-H4 (*VTCN1*) were only upregulated in SW620 CSLCs, whereas CD112 (*Nectin2*) and Galectin 3 (*LGALS3*) were upregulated in HCT-116 CSLCs (**Figures 4A, C**). Concordantly, Western blots showed higher protein levels for key checkpoint ligands in CSLCs (**Figures 4B, D**). Notably, both B7-H3 and PD-L1 expression were significantly increased in CSLCs derived from both SW620 and HCT-116 cells, relative to their parental adherent cells (cancer). On the other hand, the expression of CD155 (PVR) and CD47 proteins was significantly upregulated only in SW620 CSLCs (P < 0.01), while in HCT-116 CSLCs, CD155 was modestly decreased.

**Figure 5:**
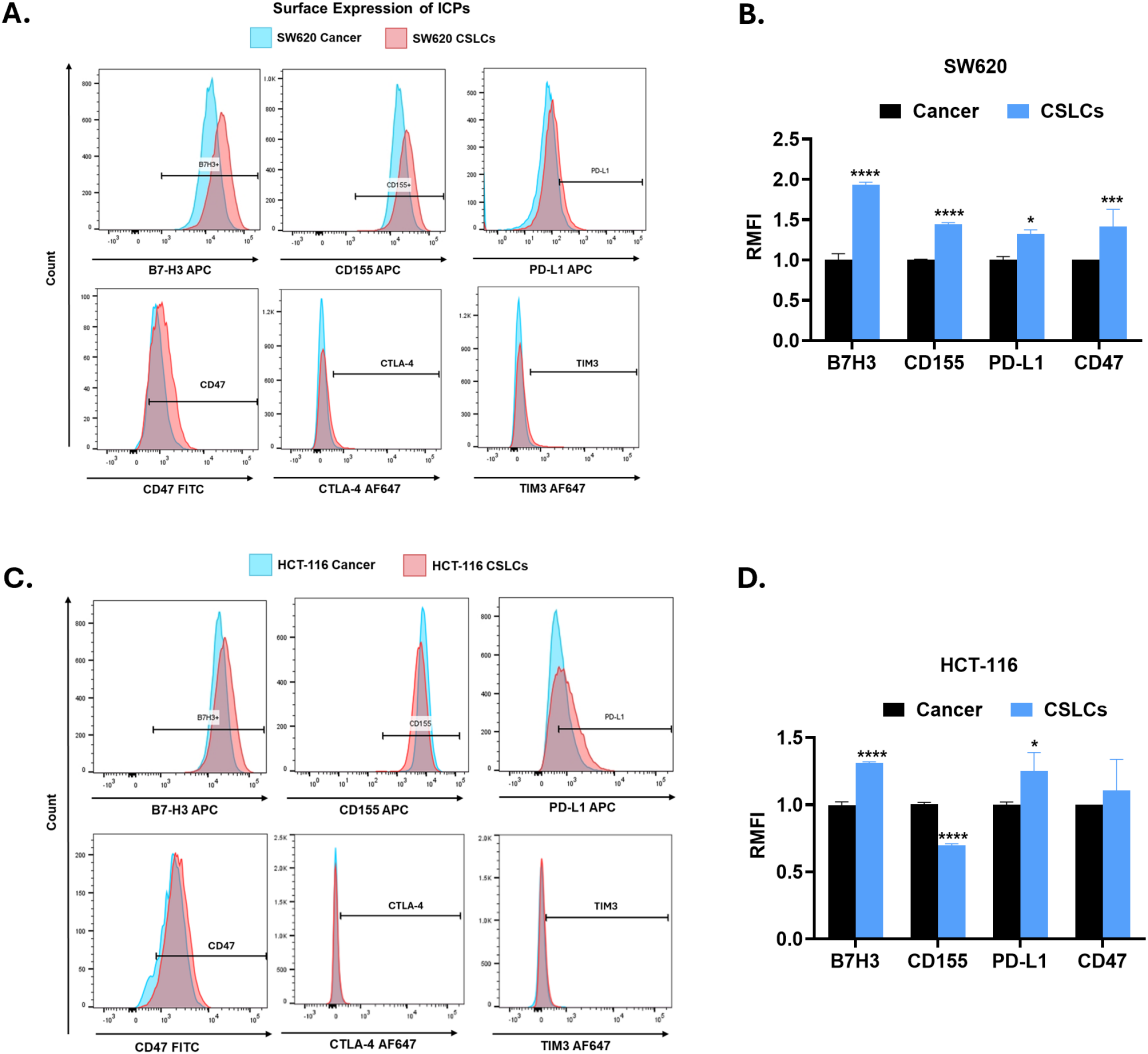
Surface expression of inhibitory immune checkpoints (ICPs) in spheroid-enriched CSLCs. **(A, C)** Representative flow cytometry histograms showing surface expression of ICP proteins in CSCs (red) and cancer (blue) for SW620 and HCT-116, respectively. **(B, D)** Relative mean fluorescence intensity (RMFI) for each ICP marker was calculated as the ratio of isotype-corrected MFI for the CSLCs to that of the paired parental cancer cells (RMFI_cancer_ = 1). Data represent mean ± SEM. *P < 0.05, **P < 0.01, ***P < 0.001, and ****P < 0.0001 by Student’s t-test; n ≥ 3.

To further validate these findings, we determined the ICP surface expression by flow cytometry (**Figure 5** and **S3**). Both cell lines were uniformly positive (~100%) for B7-H3; however, CSLCs showed higher antigen density than parental cancer cells, as indicated by right-shifted histograms, with RMFI of 1.94 (P < 0.0001) and 1.31 (P < 0.0001) in SW620 and HCT-116 CSLCs, respectively. Similarly, PD-L1 was significantly upregulated in CSLCs of both cell lines (P < 0.05), whereas CD47 showed higher expression (*p* > 0.05). On the other hand, CD155 was ~100% positive in both groups, with higher MFI in SW620 CSLCs and lower MFI in HCT-116 CSLCs as compared with cancer cells. Additionally, TIM-3 and CTLA-4 showed absent to weak surface expression by flow cytometry. Taken together, CSC-enriched spheroid cultures exhibit higher expression of multiple inhibitory ICPs, suggesting that CSCs may modulate responsiveness to ICP-targeted therapies, potentially warranting a combination approach.

### 3.4 ICP silencing modulates ALDH activity

**Figure 6:**
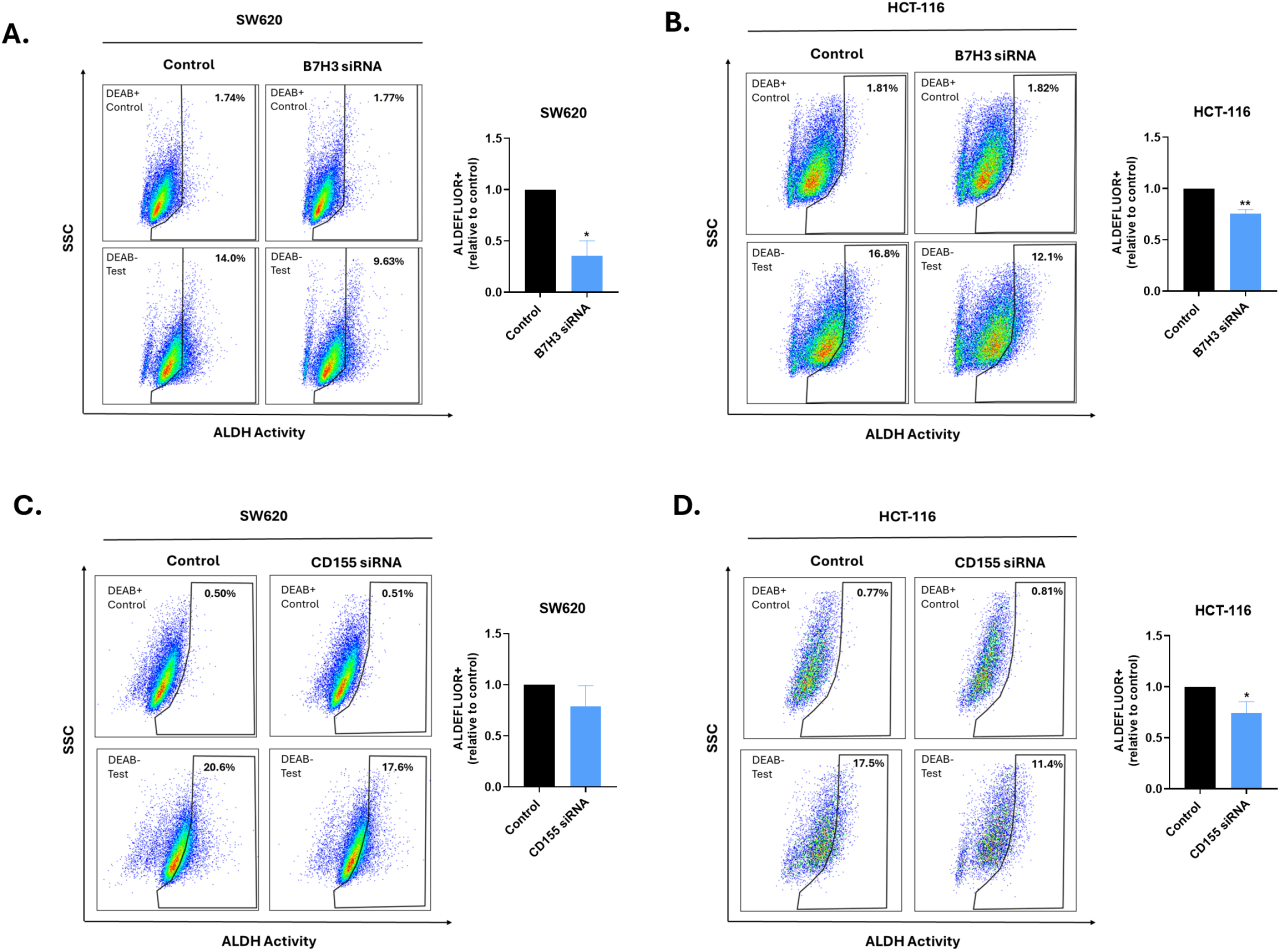
Effect of ICP silencing on ALDH activity. ALDEFLUOR™ assay of ALDH activity in adherent SW620 and HCT-116 cells following transfection with B7-H3 siRNA **(A, B)** or CD155 siRNA **(C, D)**, compared with siRNA control. Left panels show representative flow cytometry plots acquired in the presence of DEAB (+DEAB; upper) or absence of DEAB (−DEAB; lower), where DEAB serves as an ALDH inhibitor to establish baseline fluorescence and define the ALDEFLUOR™-positive gate. Right panels show quantification of ALDEFLUOR^+^ cells expressed as fold change relative to control. Data are presented as mean ± SEM (N = 3). *P < 0.05 and **P < 0.01 by Student’s t-test.

To investigate whether inhibitory immune checkpoints (ICPs) contribute to stemness, we evaluated ALDH activity using the ALDEFLUOR assay following siRNA-mediated knockdown of B7-H3 (CD276) or CD155 (PVR) (**Supplementary Figure S4**). B7-H3 silencing significantly reduced the ALDEFLUOR^+^ fraction in SW620 and HCT-116 cells by 64.3% and 24.7%, respectively (P < 0.05) (**Figures 6A, B**). On the other hand, CD155 silencing produced a modest significant decrease in ALDH activity in HCT-116 cells by 25.7% with a weaker effect observed in SW620 cells, possibly due to lower CD155 knockdown efficiency (**Figures 6C, D**).

### 3.5 Aberrant inhibitory ICP expression in CRC and its association with patient survival

**Figure 7:**
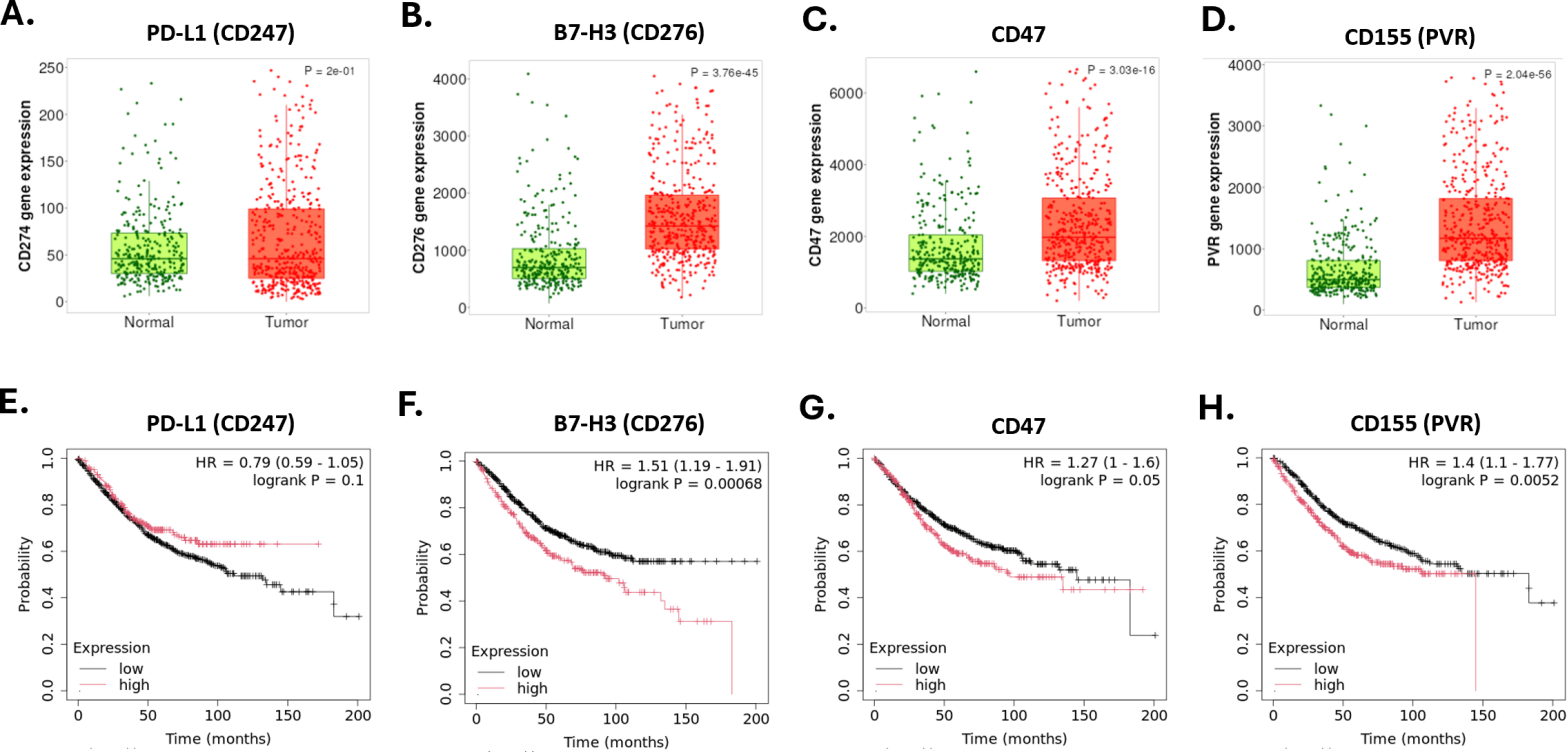
Immune checkpoint expression and association survival in colorectal cancer cohorts. **(A–D)** Box plots showing tumor vs. normal tissue expression of CD274 (PD-L1), CD276 (B7-H3), CD47, and PVR (CD155) by RNA-seq, generated with TNM_plot web tool. P values (two-sided Mann–Whitney U test) are shown. **(E-H)** The association between CD274 **(E)**, CD276 **(F)**, CD47 **(G)**, and PVR **(H)** mRNA expression and overall survival (OS) in colorectal cancer patients was analyzed by the KM plotter database (http://kmplot.com/analysis/). Colorectal cancer samples were split into high- and low-expression groups based on the median expression level, and the two patient cohorts were compared using Kaplan-Meier survival plots. The hazard ratio (HR) with 95% confidence intervals and log-rank P values were calculated.

Following our experimental validation, we assessed the potential utility of inhibitory ICPs as biomarkers in CRC. Using transcriptomic datasets from the TNM_plot platform, we compared gene expression in CRC tumors against paired adjacent normal tissue and normal colon samples from non-cancerous individuals. **Figures 7A-D** shows that CRC tumor samples exhibited a higher expression level of B7-H3 (*CD276*), CD47 and CD155 (PVR) than in normal tissues, whereas no difference in PD-L1 (*CD274*) expression between tumor and normal tissues was observed. In addition, GeneChip data accessed through the TNMplot database demonstrated that several inhibitory ICPs (e.g., CD47, CD155, PD-1, PD-L2, CTLA-4, CD200, and B7-H4) were further upregulated in metastatic tissues compared with primary tumor tissues (**Supplementary Figure S5**). However, metastatic transcriptomic data for several other ICPs genes were not available in the analyzed public dataset.

To further highlight the clinical relevance of these genes, survival analysis was performed by Kaplan–Meier Plotter. As shown in **Figures 7E-H**, high mRNA expression of B7-H3 (*CD276*) and CD155 (*PVR*) was significantly associated with poor overall survival. CD47 showed a borderline, non-significant association with poorer overall survival. On the other hand, high PD-L1 (*CD274*) expression showed a non-significant trend toward improved overall survival. These findings reinforce the biological and clinical relevance of the differential expression of these ICPs in CSCs and support their potential utility as prognostic biomarkers in CRC.

### 3.6 Correlation of inhibitory ICPs with stemness markers in CRC patients

Building on our *in vitro* CSLCs data, we next examined whether expression of inhibitory ICPs correlates with stemness markers expression in CRC patients. Notably, CD44 expression showed modest but significant positive associations with PD-L1 (*r* = 0.15; P < 0.01), B7-H3 (*r* = 0.28; P < 0.01), CD47 (*r* = 0.19; P < 0.01), and CD155 (*r* = 0.21; P < 0.01) (**Supplementary Figures S6A–D**). Nevertheless, other stemness markers showed mixed, generally weak associations (Data not shown). These findings underscore the importance of adopting a holistic, multi-marker approach to evaluate stemness in CRC.

### 3.7 mRNAsi-associated DEG and pathway enrichment

Given the absence of a single optimal stemness marker, we adopted a composite approach. We used mRNAsi, a stemness index reflecting similarity to normal stem cells derived from a one-class logistic regression (OCLR) machine-learning algorithm, for subsequent analyses (35). Samples from the TCGA COAD cohort were classified into high- and low-stemness groups based on the median mRNAsi value. Differential gene expression analysis between these two groups revealed a distinct transcriptomic divide. As illustrated in the volcano plot, numerous genes, including *DDN*, *PLA2G3*, and *MGC14436*, were significantly upregulated in the high stemness group, while genes such as *SFRP2*, *THBS4*, *CHRDL1*, and *PRELP* were downregulated considerably (**Supplementary Figure S7**).

To understand the functional implications of these changes, we performed pathway enrichment analyses of the DEGs. Gene Set Enrichment Analysis (GSEA) of Hallmark pathways showed that high-stemness tumors were strongly enriched for processes related to cell proliferation, including E2F Targets, MYC Targets V1, and G2M Checkpoint (**Supplementary Figures S7C, D**). A similar analysis using the Canonical Pathways further highlighted the activation of pathways central to genomic maintenance, such as S Phase, DNA Replication, and Homology Directed Repair (**Supplementary Figure S7D**).

Conversely, functional enrichment analysis of the downregulated genes revealed a consistent and significant suppression of pathways associated with the extracellular matrix (ECM), cell adhesion, and tissue structure. Across GO, KEGG, and Reactome databases, top downregulated pathways include Extracellular Matrix Organization, Cell Adhesion Molecules, ECM-receptor Interaction, Collagen Formation, and Focal Adhesion (**Supplementary Figure S7E**). This finding is congruent with the GSEA results, which demonstrated a relative depletion of Epithelial-Mesenchymal Transition (EMT) and Apical Junction signatures in the high-stemness group. Collectively, these molecular patterns indicate that high stemness in COAD is characterized by a dual signature: a gain of proliferative, stem-like programs and a concurrent loss of pathways that define differentiated cell identity and tissue architecture, in line with previous findings (35, 52).

The anatomical location of the tumor is known to influence prognosis of the patients and immune infiltration. To ensure that the observed associations between stemness and immune signatures were not confounded by tumor anatomical location, we analyzed the distribution of tumor site within the cohort. The proportion of patients at each anatomical site was balanced between the high- and low-stemness groups (P = 0.98) (**Supplementary Figure S8**). This confirms that the stemness-associated immune profiles identified in our study are independent of anatomical bias.

### 3.8 High stemness index correlates with an altered tumor microenvironment in CRC

**Figure 8:**
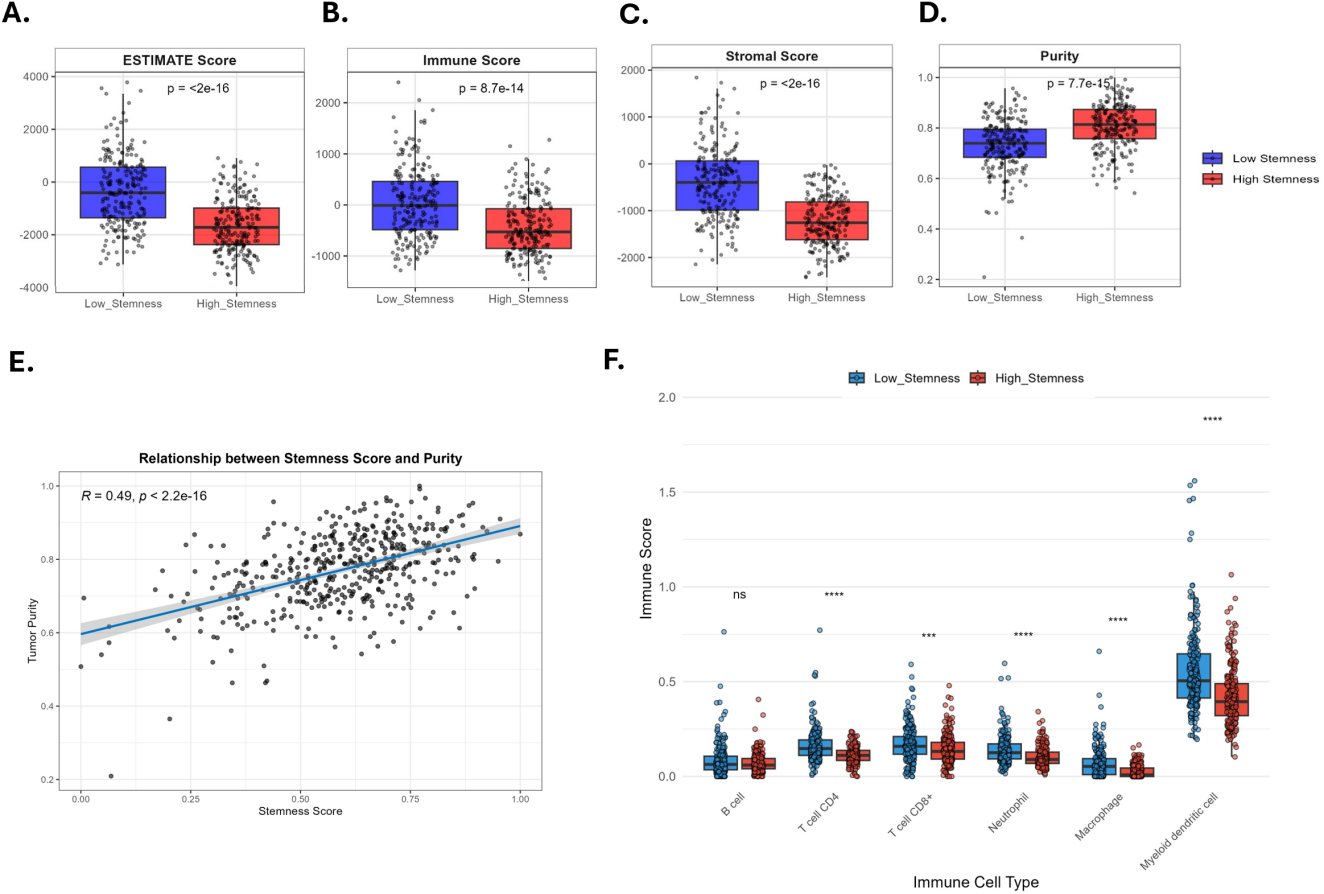
The relationship between stemness index (mRNAsi) and the tumor immune microenvironment in colorectal cancer (CRC). **(A-D)** Comparison of ESTIMATE, immune, stromal scores, and tumor purity between low- and high-stemness groups. **(E)** Correlation between mRNAsi and Tumor purity. **(F)** Comparison of tumor-infiltrating lymphocytes (TILs) between low- and high-stemness groups based on the TIMER algorithm.

We next investigated the relationship between cancer stemness and the tumor microenvironment (TME) using the ESTIMATE algorithm. This analysis revealed that tumors with high stemness had significantly lower ESTIMATE, Immune, and Stromal scores compared to their low-stemness counterparts (P < 2e-16, P = 8.7e-14, and P < 2e-16, respectively) (**Figures 8A–C**). These findings suggest that a high degree of cancer stemness is associated with reduced immune and stromal cell infiltration. In line with this, the calculated tumor purity was significantly higher in the high stemness group (p = 7.7e-15) (**Figure 8D**). Furthermore, a direct correlation analysis confirmed a significant positive relationship between the stemness score and tumor purity (R = 0.49, P < 2.2e-16), indicating that as stemness increases, the proportion of non-tumor cells in the microenvironment decreases (**Figure 8E**).

Next, we evaluated the abundance of distinct infiltrating immune and stromal cell populations between high- and low- stemness tumors using six deconvolution algorithms (xCELL, TIMER, MCPCOUNTER, quanTIseq, EPIC, and CIBERSORT). These analyses uncovered significant associations between stemness and immune infiltration (**Figure 8F** and **S9**). Myeloid dendritic cells and macrophages were generally reduced across models. In alignment with ESITMATE, cancer-associated fibroblasts and endothelial cells were lower in high-mRNAsi CRC tumors. Although differences in CD8^+^ and CD4^+^ T-cell subsets were frequently detected, the direction and magnitude were not entirely consistent across different estimation algorithms, indicating the need for additional confirmatory methods. Together, these analyses illuminate the interplay between stemness and immune–stromal composition, offering valuable insights into TME dynamics.

### 3.9 Stemness-associated variations in inhibitory checkpoint expression in CRC

**Figure 9:**
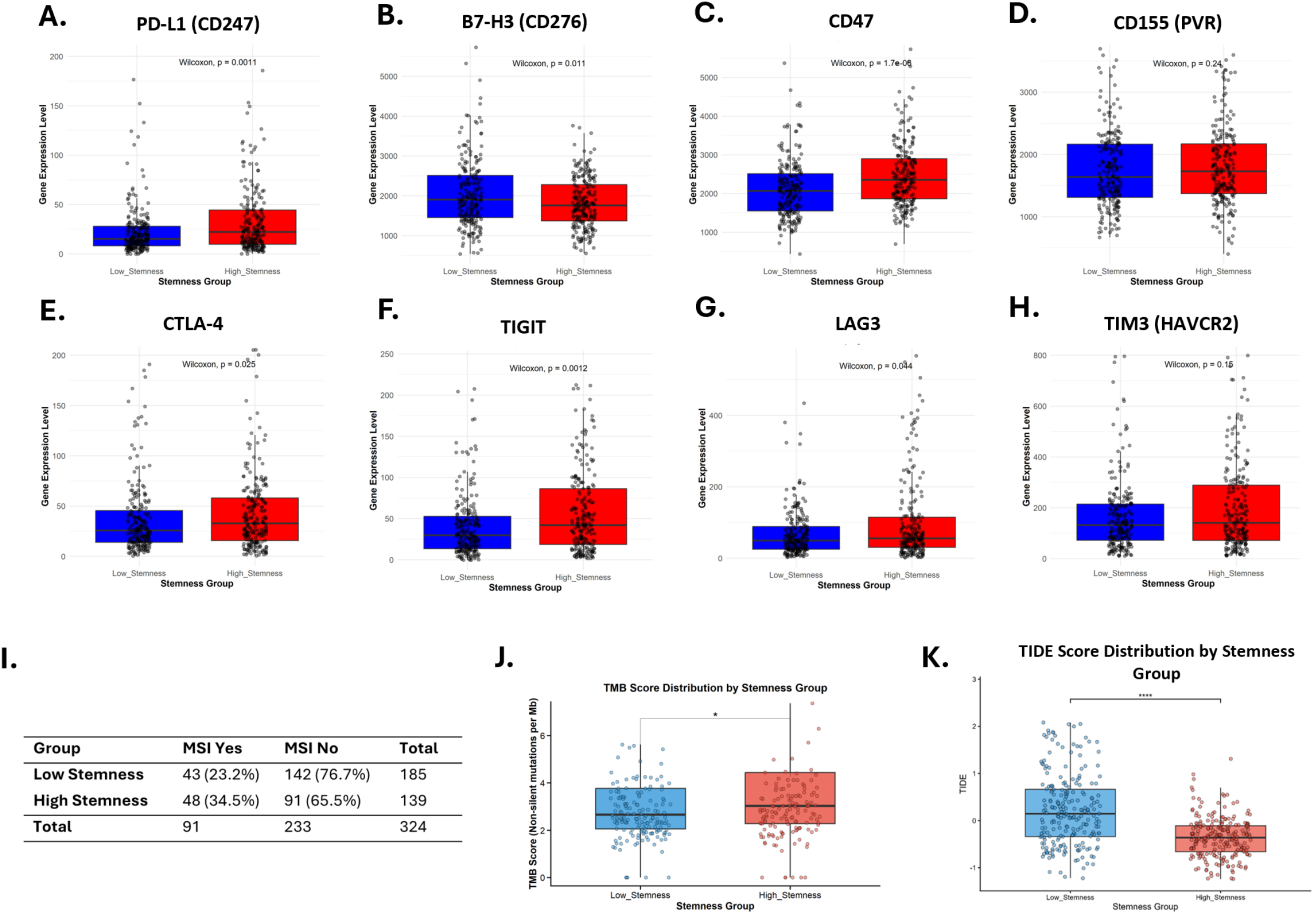
Association between cancer stemness and response to immune checkpoint inhibitors (ICIs). **(A-H)** Differential expression of Immune checkpoints (ICPs) between low stemness and high stemness groups upon adjusting for tumor purity. **(I)** Distribution of MSI status among low stemness and high stemness groups. **(J, K)** Comparison of TMB and TIDE scores between low- and high-stemness groups.

It has been well established that CSCs play a key role in mediating immune evasion, yet their interplay with inhibitory ICPs remains incompletely understood. Given the evolving role of ICIs in cancer therapy, we examined whether ICP expression varies with stemness in CRC patients. Notably, the expression of CD47 and CD155 was significantly higher in the high stemness group, whereas PD-L1 (*CD274*), B7-H3 (*CD276*), PD-1 (*PDCD1*), and CTLA-4 were lower (**Supplementary Figure S10**, **S11**). Since stemness is based on bulk RNA-seq, it can be highly influenced by heterogeneity within tumor tissues, including the presence of immune and stromal cells that also express some of these molecules, both of which were significantly lower in high-stemness tumors (**Figures 8A–E**). To evaluate the effect of this confounder, we stratified patients based on the ESTIMATE-derived purity score into three groups (low-, medium-, and high-purity). Significant variability was observed across strata. While PD-1, PD-L1, and CTLA-4 showed negative associations in the whole cohort and the high-purity stratum, analyses within low- and medium-purity strata showed positive associations (**Supplementary Figure S12**). To reduce the impact of this confounder and to obtain an overall unbiased estimate, we adjusted the mRNAsi for tumor purity (**Figures 9A–H**; **Supplementary Figure S13**, **S14**). Interestingly, after adjustment, several ICPs (e.g., PD-1, PD-L1, CD47, CTLA-4, TIM-3 and LAG3) were significantly elevated in the high stemness group. Regardless of the adjustment, B7-H3 (*CD276*) remained negatively associated with mRNAsi score, whereas CD47 remained positively associated. Taken together, these findings reveal distinct ICP expression profiles in CSCs, underscoring the importance of accounting for CSCs when designing checkpoint-based therapeutic strategies and predicting treatment responses.

### 3.10 Correlation between stemness and response to immune checkpoint inhibitors

Given that a high stemness index is associated with an altered TME, we sought to explore its connection to established biomarkers of response to ICI therapy. Microsatellite instability (MSI) is a classic predictor of ICI efficacy in CRC. Our analysis revealed that MSI status distribution differed significantly between the high- and low-stemness groups (χ² = 5.92, P = 0.015), suggesting a link between stemness and this key clinical biomarker (**Figure 9I**).

Furthermore, we assessed the Tumor Mutation Burden (TMB), another commonly reported biomarker for ICI response. The high-stemness group exhibited a significantly higher TMB than the low-stemness group (P < 0.05) (**Figure 9J**). A high TMB is an established predictor of a favorable response to ICIs, as a greater number of mutations increases the likelihood of producing immunogenic neoantigens, that can be targeted by the immune system following checkpoint blockade.

To obtain an integrated prediction of ICI sensitivity, we applied the TIDE algorithm. Consistent with the TMB findings, the TIDE prediction score was significantly lower in the high-stemness group (P < 0.0001), suggesting a greater predicted likelihood of response to ICI therapy in these tumors (**Figure 9K**). While these findings collectively indicate that high-stemness tumors possess molecular features associated with improved ICI response, it is essential to note that the TIDE algorithm was not specifically developed or validated for CRC (47).

## 4 Discussion

Immune checkpoint blockade has emerged as a key pillar in cancer therapy, yielding specific and durable antitumor responses. In particular, inhibitors of PD-1 (e.g., nivolumab) and CTLA-4 (e.g., ipilimumab) have shown remarkable success in several malignancies, including non-small-cell lung cancer and melanoma. However, only a minority of CRC patients with dMMR/MSI respond to these therapies (53). The limited efficacy could be partially attributed to heterogeneity within the TME.

Emerging evidence suggests that CICs/CSCs mediate immune evasion by differentially expressing ICPs, modulating checkpoint downstream signaling, or fostering an immunosuppressive TME (36). To date, it remains unclear whether current ICIs can effectively eliminate CSC subpopulations. Moreover, the full array of ICP ligands expressed by CSCs remains unknown. Given the importance of CSCs in driving tumor recurrence, metastasis, immune evasion, and therapeutic resistance, it is crucial to delineate their checkpoint landscape. Therefore, our study fills a critical gap by systematically profiling a panel of ICPs in colorectal cancer and CSLCs.

The 3D tumor-spheres culture is widely used to enrich putative stem-like cells from solid tumors (54–56). The resulting cell populations display phenotypic and functional properties similar to those of stem cells. In the current study, our CSC-enriched spheroid cultures (CSLCs) featured upregulation of a wide range of stemness-associated regulators and surface markers as compared to their parental adherent cells (cancer), such as Nanog, SOX9, β-catenin and EpCAM, which were more pronounced in SW620 than in HCT-116, aligning with the higher invasive potential of SW620 (57), a feature closely associated with CSC phenotypes (58). The link between these markers and stemness has been validated in multiple studies (59–63). On the other hand, CD44, CD133, and CD166 were not uniformly upregulated across the cell lines and between mRNA and protein levels. While several studies have reported enhanced stemness associated with CD166, CD133, or CD44 expression in CRC (54, 64, 65), other studies have highlight their inconsistent performance across models, questioning their utility as a universal stemness marker in CRC (66, 67). Moreover, ALDH expression was significantly upregulated at mRNA and protein levels in CSLCs of both cell lines. Nevertheless, its activity, as measured by the ALDEFLUOR assay, was upregulated only in SW620. Similarly, Khorrami et al. reported lower ALDH activity in HT-29 spheroids despite their higher *in vivo* tumorigenic potential (68). A plausible explanation for the lower ALDEFLUOR signal in some CSC subpopulations is the upregulation of efflux transporters (e.g., ABCG2/ABCB1) (69, 70), which can pump out ALDEFLUOR substrate or product. Therefore, incomplete blockade of these transporters may underestimate actual ALDH activity (71).

These findings show that CSCs comprise highly heterogeneous subpopulations and relying on a single stemness marker for isolation of CSCs may not capture the full CSC spectrum. Therefore, in this study, we utilized spheroid culture rather than marker-based cell sorting to enrich diverse CSC subpopulations. Accordingly, the upregulation of multiple stemness markers suggests that we captured at least part of this heterogeneity. Although we did not perform *in vivo* experiments to directly evaluate the enhanced tumorigenicity of our CSC-enriched cultures, prior studies using colorectal cancer spheroids have shown that these populations exhibit greater tumor-initiating capacity *in vivo* (68, 72, 73).

Upon demonstrating that CSC-enriched spheroid cultures (CSLCs) exhibited induction of several stemness markers, we next examined whether these stem-like states were accompanied by differential expression of immune checkpoint ligands. To the best of our knowledge, this is the first extensive analysis of ICP expression in colorectal CSCs. We focused on checkpoint ligands that are known to have immunosuppressive roles in the TME. Our data revealed that both cancer cells and CSLCs express multiple ligands, but CSLCs displayed higher levels of several key checkpoints. Remarkably, our findings showed, for the first time, that B7-H3 was elevated in CSLCs derived from SW620 and HCT-116 cell lines, with notable upregulation on the cell surface, accompanied by a proportional increase in stemness markers. In this context, previous studies showed a significant association between B7-H3 and promotion of cell invasion and metastasis via EMT and increased the expression of stemness marker (i.e, CD133, CD44, and OCT4), whereas B7-H3 knockdown in Caco-2 cells led to opposite effects (74, 75).

Additionally, we showed that PD-L1 was significantly upregulated in CSLCs of both cell lines, which was consistent with Wei et al.’s observations in HCT-116 and HT-29 (76). While PD-L1 is typically recognized for suppressing anti-tumor immune responses, recent studies have suggested that PD-L1 is also involved in EMT, metastasis, therapy resistance, and stemness (77). Mechanistically, PD-L1 has been reported to activate HMGA1-dependent signaling, including the PI3K/Akt and MEK/ERK pathways, thereby expanding the CSC pool and enhancing tumorigenicity *in vivo* (76). Moreover, elevated expression of the stemness marker (Ly6K) in breast cancer tissue has been reported to correlate with higher levels of PD-L1. Notably, Lanuza et al. observed higher PD-L1 expression in spheroid cultures of Caco-2 and HT-29 but not in HCT-116 (78), highlighting potential cell line–specific differences in checkpoint regulation.

Despite its pro-tumorigenic roles, the KM-plotter analysis showed a non-significant trend toward better prognosis with higher PD-L1 (CD274) gene expression in CRC. This apparent discrepancy likely reflects the difference between tumor cell–intrinsic PD-L1 upregulation in CSC-enriched cultures and PD-L1 measured in bulk tumors, where the signal represents a mixture of tumor, stromal, and immune cells. In addition, in immune-inflamed tumors, PD-L1 can be induced by IFN-γ released from activated CD8^+^ T cells as part of an adaptive immune resistance response (79). Thus, elevated PD-L1 may reflect an ongoing anti-tumor immune response rather than intrinsic tumor aggressiveness (80, 81). Consistently, T-cell–inflamed gene expression signatures have been shown to correlate with PD-L1 expression and improved clinical outcomes in multiple cancers (79). In this line, a meta-analysis by Alexander et al. suggested that PD-L1 expression on immune cells is associated with favorable prognosis, whereas its expression on tumor cells yielded heterogeneous outcomes (82).

CD155 mediates immunosuppression by engaging with TIGIT on T cells and NK cells, thereby dampening T cell and NK cell-mediated cytotoxicity (83, 84). The upregulation of CD155 in a subset of CRC CSLCs observed in this study is a new finding. In line with our findings, CD155 has been reported to be elevated in primary glioma CSCs (38) and osteosarcoma CSC-enriched spheroids (85), whereas CD44^+^CD24^-/low^ breast CSCs display CD155 levels comparable to those of non-CSCs (86). Although its role in CRC CSCs has not yet been established, CD155 has been shown to enhance stemness in osteosarcoma by activating Wnt/β-catenin signaling via the SRC/AKT/GSK3β axis (85). Interestingly, CD155 (*PVR)*-targeted chimeric antigen receptor T (CAR T) cells exhibited antitumor activity against glioma stem cells and in xenograft models (87). These findings suggest that CD155 is a promising therapeutic target for enhancing the elimination of CSCs in CRC.

We also found that CD47 is higher in CSLCs compared with bulk cancer cells, suggesting that it may promote CSC-mediated immune evasion, enabling them to escape macrophage phagocytosis. Prior to this work, direct evidence for differential CD47 expression in CRC CSCs was lacking. However, Fujiwara-Tani et al. reported an association between CD44 and CD47 expression in CRC tissues and that their co-expression correlated with increased metastasis, poor survival, and potential resistance to nivolumab (88). The concurrent upregulation of stemness-associated genes and ICPs suggests a possible link between the stem-like phenotype and ICP-mediated immune evasion.

Similar to the trend observed for stemness markers, SW620 spheroids exhibited a more pronounced upregulation of ICPs than HCT-116 spheroids, which is consistent with the higher invasive phenotype of SW620. In line with these findings, TNMplot analysis showed that CD155 and CD47 expression were significantly elevated in metastatic colon tissue compared with non-metastatic and normal tissues. The observation that not all checkpoint ligands are uniformly upregulated, and that the set of altered ligands differs between the two cell lines, reinforces the presence of intra-CSC heterogeneity. This suggests that distinct CSC subpopulations may employ different sets of inhibitory checkpoints to evade immune surveillance. Such heterogeneity may have significant therapeutic and prognostic implications. Therefore, future studies employing multiparametric co-staining approaches to assess the co-expression of stemness markers and ICPs will help delineate immunomodulatory programs across distinct CSC subpopulations. For instance, Tout et al. showed that cell subsets co-expressing ICPs (e.g., PD-L1^+^PD-L2^+^ or PD-L1^+^CTLA-4^+^) together with stemness markers (i.e., CD44v6, LGR5, and OCT4) were enriched in primary colorectal CSCs compared with differentiated tumor counterparts (89). Nevertheless, further studies employing expanded multiparametric panels that incorporate additional stemness markers and immune checkpoints are warranted.

Given the limited proportion of CRC patients benefiting from PD-1/PD-L1 therapy, our findings underscore the importance of expanding beyond conventional checkpoints and considering CSC-upregulated ICPs as potential biomarkers and therapeutic targets. Upregulation of co-inhibitory receptors (e.g., LAG3, TIM3, TIGIT) or their ligands has been described as a compensatory mechanism of resistance to ICIs (90–92). For instance, non-responders to anti-PD-1 or combined anti-PD-1/anti-CTLA-4 therapy were found to exhibit distinct expression patterns of alternative ICPs (91). Likewise, high pretreatment CD155 expression has been associated with poor response to anti-PD-1 therapy in melanoma patients (93). Accordingly, combined targeting of multiple checkpoints (e.g., PD-1/TIM3 or PD-1/TIGIT/CD155) may enhance antitumor responses and help overcome adaptive resistance (94–96). Mechanistically, CD155 can suppress the activity and migration of CD8^+^ T cells through the PI3K/AKT/NF-κB pathway in CRC preclinical models (97). Likewise, in NSCLC, B7-H3 expression was associated with nonresponse to anti-PD-1 therapy, and dual-blockade of B7-H3 and PD-L1 enhanced antitumor immunity (98). Therefore, monotherapy as a means of countering checkpoint-mediated immune suppression is unlikely to achieve optimal antitumor effects. Rational, personalized combinations of ICP inhibitors, particularly targeting those upregulated in CSCs, may improve therapeutic responses and reduce recurrence by eliminating residual CSCs.

Although clinical evidence remains limited, preclinical studies indicate that blocking ICPs overexpressed by CSCs, such as B7-H3 or CD47, can diminish CSC populations (99–102). A recent study by Chen et al. showed that blockade of CD155 could suppress stemness of osteosarcoma via the SRC/β-catenin signaling axis (85). However, direct evidence for a comparable link in CRC is still lacking. Our data provides initial support for such connection, where the knockdown of B7H3 and CD155 resulted in a significant decrease in ALDH activity. Nevertheless, further work is required to define the molecular mechanisms downstream of each differentially expressed ICP and to determine whether these effects translate to *in vivo* tumor initiation/maintenance and to immune-dependent antitumor activity.

Analysis of publicly available CRC RNA-seq datasets further supports the clinical relevance of our *in vitro* observations. These findings underscore the potential of ICPs not only as therapeutic targets but also as prognostic biomarkers. Given emerging evidence that CSC frequency and stemness indices reflect immunological states, considering CSC burden as a biomarker may aid in patient selection for immunotherapy.

In the TCGA-COAD cohort, high-stemness tumors showed reduced immune and stromal infiltration, yet exhibited higher TMB, increased MSI frequency, elevated ICP expression after adjustment for tumor purity, and lower TIDE scores. These findings suggest that tumors with high stemness indices, despite their immunosuppressive TME, are associated with molecular traits that could sensitize them to immune checkpoint blockade. Nevertheless, this paradox should be interpreted cautiously, as tumors with low TILs often exhibit limited responses to ICIs (103, 104). Moreover, given the upregulation of multiple ICPs, these observations provide a rationale to explore combined ICI approaches as a potential strategy to improve therapeutic responses. Further experimental studies are warranted to determine whether CSCs can be effectively targeted by ICIs, and whether combination strategies could enhance therapeutic efficacy. Supporting this, several preclinical models, including those combining ICIs with CSC-targeted therapies such as BMI1 inhibition (105), ALDH^high^ CSC lysate pulsed dendritic cell vaccines (106), and c-MET blockade (107), have already shown promise in depleting CSCs (36).

Given the critical role of ICPs in shaping tumor-immune interactions, future *in vivo* studies are warranted to determine whether the altered ICP profile observed in stem-like cells is maintained within the TME and to assess its impact on immune evasion and response to immunotherapy. We acknowledge that the clinical analyses in this study rely on bulk RNA-seq data from TCGA. While we adjusted for tumor purity to mitigate the confounding effects of non-tumor cells, bulk sequencing ultimately represents an average of all cell types within the tumor microenvironment. Consequently, the mRNAsi reflects the aggregate phenotype of the tissue rather than isolated CSCs. However, these *in silico* findings are strongly supported by our *in vitro* data, where we observed direct upregulation of ICPs in CSC-enriched spheroids compared to parental cells. This dual approach validates that, while bulk RNA-seq has limitations, the upregulation of ICPs is likely an intrinsic trait of colorectal CSLCs. Future studies utilizing single-cell RNA sequencing (scRNA-seq) would be valuable to further dissect the precise cellular sources of these immune checkpoints in the clinical setting.

## 5 Conclusion

Taken together, our findings demonstrate that colorectal CSLCs exhibit a distinct immunomodulatory profile, characterized by altered expression of multiple inhibitory ICPs. In parallel, bioinformatic analysis of publicly available CRC transcriptomic datasets (TCGA) suggests that higher stemness features associate with reduced immune infiltration and with genomic/immunogenic characteristics including increased TMB/MSI, supporting that CSC-related programs may modulate responsiveness to immune checkpoint inhibitors (ICIs).

By integrating *in vitro* CSC-enrichment experiments with TCGA-based transcriptomic analyses, our study highlights colorectal CSCs as promising therapeutic targets and supports their potential utility as biomarkers to guide checkpoint-based immunotherapy strategies in colorectal cancer. Future work should define the functional roles of these checkpoints in CSC-immune cell crosstalk, validate their predictive value for ICI responsiveness in clinical cohorts and evaluate rational combination regimens combining CSC-directed therapies with multi-checkpoint blockade. Collectively, these findings underscore the need to view CSCs not only as drivers of recurrence and therapy resistance but also as potential predictive biomarkers and therapeutic targets for personalized immunotherapy in colorectal cancer.

## Data Availability

Publicly available datasets were analyzed in this study. This data can be found here: TCGA COAD https://portal.gdc.cancer.gov/.

## Supplementary material

Supplementary Figure 1 Expression of stemness-associated markers by RT-qPCR in CSC-enriched spheroids versus bulk cancer cells. RT-qPCR analysis of stemness-associated genes across different passages of HT-29 **(A)** and SW480 **(B)** spheroid cultures (CSCs) compared with their respective parental adherent (cancer) cells. mRNA levels were normalized to the housekeeping gene GAPDH and are shown as fold change relative to cancer cells. Data represent mean ± SEM. *P < 0.05, **P < 0.01, and ***P < 0.001 by Student’s t-test; n ≥ 3.

Supplementary Figure 2 Surface expression of stemness-associated markers in spheroid-enriched CSLCs. Representative flow-cytometry histograms of selected stemness markers in spheroid-enriched cancer stem–like cells (CSLCs; red) compared with the corresponding adherent/parental cancer cells (blue) in **(A)** HCT-116 and **(B)** SW620. Gray histograms indicate the matched isotype control (for directly conjugated antibodies) or secondary-antibody–only control (for unconjugated primary antibodies).

Supplementary Figure 3 Surface expression of selected immune checkpoint (ICPs) in spheroid-enriched CSLCs. Representative flow-cytometry histograms of selected ICPs in spheroid-enriched cancer stem–like cells (CSLCs; red) compared with the corresponding adherent/parental cancer cells (blue) in **(A)** HCT-116 and **(B)** SW620. Gray histograms indicate the matched isotype control (for directly conjugated antibodies) or secondary-antibody–only control (for unconjugated primary antibodies).

Supplementary Figure 4 Validation of B7-H3 and CD155 silencing by siRNA using flow cytometry. Representative overlaid histograms showing surface expression of B7-H3 **(A)** and CD155 **(B)** in control cells (blue) compared with cells transfected with specific siRNA (red).

Supplementary Figure 5 Box plots showing normal vs. Tumor vs. metastatic colon tissue expression of **(A)** CD47, **(B)** CD155, **(C)** PD-L2 by Gene chip data, generated with TNM_plot web tool. P values (Kruskal-Wallis P) are shown.

Supplementary Figure 6 **(A-D)** Spearman correlation between CD44 expression (y-axis) and selected immune checkpoints (x-axis) in colorectal cancer patients generated using TNM_plot (https://tnmplot.com/analysis/, accessed on September 5, 2025).

Supplementary Figure 7 Identification of differentially expressed genes (DEGs) and their functional characterization across high- and low- stemness groups. **(A)** Volcano plot showing DEGs between high-stemness and low-stemness groups in the TCGA colorectal cancer cohort, classified based on the median mRNAsi score. Genes upregulated in high-stemness group are shown in red, significantly downregulated genes in blue, and non-significant genes in grey, using |log2FC| > 1 and P < 0.05 as cut off criteria. Top-ten highly dysregulated genes are labeled. **(B)** Top DEGs ranked by log2 fold change and statistical significance, highlighting the most significantly upregulated and downregulated genes in high-stemness tumors. **(C, D)** Gene Set Enrichment Analysis (GSEA) of Hallmark and Reactome pathways showing pathways positively enriched in high-stemness tumors (positive NES) and pathways enriched in low-stemness tumors (negative NES). **(E)** Functional enrichment analysis of genes downregulated in high-stemness tumors using GO Biological Process, KEGG, and Reactome databases. Dot size corresponds to gene count and color reflects adjusted P value.

Supplementary Figure 8 Distribution of anatomical site of tumor in high stemness and low stemness groups.

Supplementary Figure 9 Comparison of tumor-infiltrating lymphocytes (TILs) between low- and high-stemness groups based on the CIBERSORT **(A)**, EPIC **(B)**, MCPCOUNTER **(C)**, QUANTISEQ **(D)**, XCELL **(E)** algorithms.

Supplementary Figure 10 Correlation between non-corrected mRNAsi and the expression of selected inhibitory immune checkpoints (ICPs).

Supplementary Figure 11 Differential expression of Immune checkpoints (ICPs) between low stemness and high stemness groups stratified based on non corrected mRNAsi score.

Supplementary Figure 12 **(A)** Log2 fold-change of inhibitory immune checkpoint genes between high- and low-stemness tumors across tumor-purity strata. Horizontal bar plots show the log2 fold-change (high-stemness vs. low-stemness) for each immune checkpoint gene within the TCGA CRC cohort, stratified by tumor purity (high-purity, medium-purity, and low-purity groups), alongside the whole-cohort analysis. **(B)** Correlation between inhibitory immune checkpoint expression and stemness score across tumor-purity strata. Bar plots display the correlation coefficients between each immune checkpoint gene and the mRNAsi stemness score within the TCGA CRC cohort. Analyses were stratified by tumor purity (high-, medium-, and low-purity groups), with the whole-cohort correlation shown for comparison.

Supplementary Figure 13 Correlation between tumor-purity adjusted mRNAsi and the expression of selected inhibitory immune checkpoints (ICPs).

Supplementary Figure 14 Differential expression of Immune checkpoints (ICPs) between low stemness and high stemness groups stratified based on tumor-purity adjusted mRNAsi score.
