# Supplementary material for: Expression profiling of inhibitory immune checkpoints in colorectal cancer stem cells and their association with tumor immunity and immunotherapy biomarkers
Source: Front Immunol. 2026 Apr 8;17:1760555. doi: 10.3389/fimmu.2026.1760555 (PMC13100989; doi:10.3389/fimmu.2026.1760555)
Supplement: Supplementary Figure 1 — Expression of stemness-associated markers by RT-qPCR in CSC-enriched spheroids versus bulk cancer cells. RT-qPCR analysis of stemness-associated genes across different passages of HT-29 (A) and SW480 (B) spheroid cultures (CSCs) compared with their respective parental adherent (cancer) cells. mRNA levels were normalized to the housekeeping gene GAPDH and are shown as fold change relative to cancer cells. Data represent mean ± SEM. *P < 0.05, **P < 0.01, and ***P < 0.001 by Student’s t-test; n ≥ 3. [file Table1.docx]

Supplementary Material

# Supplementary Tables

Table S1 RT-qPCR primers used in the present study

| Gene | Primer sequences (5’-3’) |
| --- | --- |
| CD44 | Forward primer: GGAGCAGCACTTCAGGAGGTTAC |
|  | Reverse primer: GGAATGTGTCTTGGTCTCTGGTAGC |
| CD44v6 | Forward primer: CCAGGCAACTCCTAGTAGTACAACG |
|  | Reverse primer: CGAATGGGAGTCTTCTTTGGGT |
| CD133 | Forward primer: AAGCATTGGCATCTTCTATGG |
|  | Reverse primer: AAGCACAGAGGGTCATTGAGA |
| CD166 | Forward primer: AGTCTTCATTATCAGGATGC |
|  | Reverse primer: GGGATCAGTTTTCTTTGTCA |
| EPCAM | Forward primer: GCTGGTGTGTGAACACTGCT |
|  | Reverse primer: ACGCGTTGTGATCTCCTTCT |
| ALDH1A1 | Forward primer: ACTGCTCTCCACGTGGCATCTTTA |
|  | Reverse primer: TGCCAACCTCTGTTGATCCTGTGA |
| Nanog | Forward primer: ATGCCTCACACGGAGACTGT |
|  | Reverse primer: AAGTGGGTTGTTTGCCTTTG |
| SOX4 | Forward primer: GGTCTCTAGTTCTTGCACGCTC |
|  | Reverse primer: CGGAATCGGCACTAAGGAG |
| SOX9 | Forward primer: ATGAACGCCTTCATGGTGT |
|  | Reverse primer: TCTCGCTCTCGTTCAGAAGT |
| OCT4 | Forward primer: TCGAGAACCGAGTGAGAGG |
|  | Reverse primer: GAACCACACTCGGACCACA |
| c-Myc | Forward primer: GGAACGAGCTAAACGGAGCT |
|  | Reverse primer: GGCCTTTTCATTGTTTTCCAATT |
| PD-1 | Forward primer: CGTGGCCTATCCACTCCTCA |
|  | Reverse primer: ATCCCTTGTCCCAGCCACTC |
| PDL-1 | Forward primer: AAATGGAACCTGGCGAAAGC |
|  | Reverse primer: GATGAGCCCCTCAGGCATTT |
| B7-H3 | Forward primer: CTGGCTTTCGTGTGCTGGAGAA |
|  | Reverse primer: GCTGTCAGAGTGTTTCAGAGGC |
| B7-H4 | Forward primer: AGGGAGTGGAGGAGGATACAG |
|  | Reverse primer: GCAGCAGCCAAAGAGACAG |
| CD47 | Forward primer: AGAAGGTGAAACGATCATCGAGC |
|  | Reverse primer: CTCATCCATACCACCGGATCT |
| CD112 | Forward primer: CGGAACTGTCACTGTCACCA |
|  | Reverse primer: GACACTTCAGGAGGGTAGCG |
| CEACAM1 | Forward primer: TCTACCCTGAACTTTGAAGCCCA |
|  | Reverse primer: TGAGAGACTTGAAATACATCAGCACTG |
| Galectin 3 | Forward primer: ATGCAAACAGAATTGCTTTAGATT |
|  | Reverse primer: AGTTTGCTGATTTCATTGAGTTTT |
| HVEM | Forward primer: GTGCAGTCCAGGTTATCGTGT |
|  | Reverse primer: CACTTGCTTAGGCCATTGAGG |

Table S2 List of primary and secondary antibodies used in this study for Western blot (WB)

| Antibody | Clone | Host/Isotype | Company | Catalog Number | Dilution | Molecular Weight |
| --- | --- | --- | --- | --- | --- | --- |
| HCAM (CD44) | F-4 | Mouse IgG1 κ | Santa Cruz | sc-9960 | 1:200 | 90-05 kDa |
| Anti-CD44v6 [VFF-7] | VFF-7 | Mouse IgG1 | Abcam | ab30436 | 1:1000 | 80 kDa |
| CD133 | A3G6K | Rabbit IgG | Cell Signaling | 5860S | 1:1000 | 133 kDa |
| Anti-CD166 | EPR2759(2) | Rabbit IgG | Abcam | ab109215 | 1:1000 | Pred. 65 kDa Obs. 100 kDa |
| EpCAM | VU1D9 | Mouse IgG1 | Cell Signaling | 2929S | 1:1000 | 40 kDa |
| ALDH1A1 | B-5 | Mouse IgG2a κ | Santa Cruz | sc-374149 | 1:100 | 56 kDa |
| Nanog | 1E6C4 | Mouse IgG1 κ | Santa Cruz | sc-293121 | 1:200 | 40 kDa |
| Sox2 XP® | D6D9 | Rabbit | Cell Signaling | 3579S | 1:1000 | 35 kDa |
| Sox9 | D8G8H | Rabbit IgG | Cell Signaling | 82630S | 1:1000 | 70 kDa |
| Anti-LGR5 | EPR3065Y | Rabbit IgG | Abcam | ab75850 | 1:1000 | 100 kDa |
| β-catenin | E-5 | Mouse IgG1 κ | Santa Cruz | Sc-7963 | 1:200 | 92 kDa |
| B7-H3 XP® | D9M2L | Rabbit IgG | Cell Signaling | 14058S | 1:1000 | 90 kDa |
| PD-L1: Pdcd-1L1 | 1C10 | Mouse IgG2b κ | Santa Cruz | sc-293425 | 1:200 | Pred. 33 kDa Obs. 47 kDa |
| PVR/CD155 | D8A5G | Rabbit IgG | Cell Signaling | 81254S | 1:1000 | 60-80 kDa |
| CD47 | B6H12 | Mouse IgG1 κ | Santa Cruz | sc-12730 | 1:100 | 47-60 kDa |
| B7-H4 XP® | D1M8I | Rabbit IgG | Cell Signaling | 14572S | 1:1000 | 75 kDa |
| GAPDH | 14C10 | Rabbit | Cell Signaling | 2118S | 1:1000 | 37 kDa |
| α-Tubulin | EP1332Y | Rabbit IgG | Abcam | ab52866 | 1:25,000 | 50 kDa |
| Anti-mouse IgG, HRP-linked | Polyclonal | Horse | Cell Signaling | 7076 | 1:1000-1:3000 | N/A |
| Anti-rabbit IgG, HRP-linked Antibody | Polyclonal | Goat | Cell Signaling | 7074 | 1:1000-1:3000 | N/A |
| m-IgGκBP-HRP | Recombinant | N/A | Santa Cruz | sc-516102 | 1:1000-1:3000 | N/A |

Table S3 List of primary and secondary antibodies used in this study for Flow cytometry

| Antibody | Clone | Host/Isotype | Company | Catalog Number | Dilution |
| --- | --- | --- | --- | --- | --- |
| APC anti-human CD276 (B7-H3) Antibody | MIH42 | Mouse IgG1, κ | BioLegend | 351005  Lot: B429472 | 1:40 |
| APC anti-human CD155 (PVR) | SKII.4 | Mouse IgG1, κ | BioLegend | 337617  Lot B439768 | 1:160 |
| APC anti-human CD274 (B7-H1, PD-L1) | MIH3 | Mouse IgG1, κ | BioLegend | 374513  Lot: B364974 | 1:20 |
| CD47 | B6H12 | Mouse IgG1, κ | Santa Cruz | sc-12730 | 1:20 |
| CTLA-4 | F-8 | Mouse IgG1 κ | Santa Cruz | sc-376016 | 1:20 |
| TIM-3 | D5D5R | Rabbit IgG | Cell Signaling | 45208 | 1:50 |
| CD24 ECD | ALB9 | Mouse IgG1 | Beckman Coulter | B12699 | 1:80 |
| EpCAM | VU1D9 | Mouse IgG1 | Cell Signaling | 2929S | 1:800 |
| APC Mouse IgG1, κ Isotype Ctrl (FC)  Antibody | MOPC-21 | Mouse IgG1, κ | BioLegend | 400122  Lot: B444428 | same concentration of the primary antibody |
| Goat anti-Mouse IgG (H+L) Cross-Adsorbed Secondary Antibody, Alexa Fluor™ 647 | Polyclonal | Goat IgG | ThermoFischer Scientific | A-21235 | 1:400-1:800 |
| Goat anti-Rabbit IgG (H+L) Cross-Adsorbed Secondary Antibody, Alexa Fluor™ 647 | Polyclonal | Goat IgG | ThermoFischer Scientific | A-21244 | 1:400 |
| Rabbit anti-Mouse IgG (H+L) Cross-Adsorbed Secondary Antibody, Alexa Fluor™ 488 | Polyclonal | Rabbit IgG | ThermoFischer Scientific | A-11059 | 1:400 |

Table S4 List of primary and secondary antibodies used in this study for immunofluorescence (IF)

| Antibody | Clone | Host/Isotype | Company | Catalog Number | Dilution |
| --- | --- | --- | --- | --- | --- |
| ALDH1A1 | B-5 | Mouse IgG2a κ | Santa Cruz | sc-374149 | 1:50 |
| SOX9 | D8G8H | Rabbit IgG | Cell Signaling | 82630S | 1:50 |
| Nanog | 1E6C4 | Mouse IgG1 κ | Santa Cruz | sc-293121 | 1:50 |
| Cytokeratin Pan Type I | AE1 | Mouse IgG1 | ThermoFischer Scientific | MA5-13144 | 1:100 |
| Goat anti-Mouse IgG (H+L) Cross-Adsorbed Secondary Antibody, Alexa Fluor™ 594 | Polyclonal | Goat IgG |  | A11005 | 1:500-1:1000 |
| Goat anti-Rabbit IgG (H+L) Cross-Adsorbed Secondary Antibody, Alexa Fluor™ 594 | Polyclonal | Goat IgG |  | A11012 | 1:1000 |

# Supplementary Figures


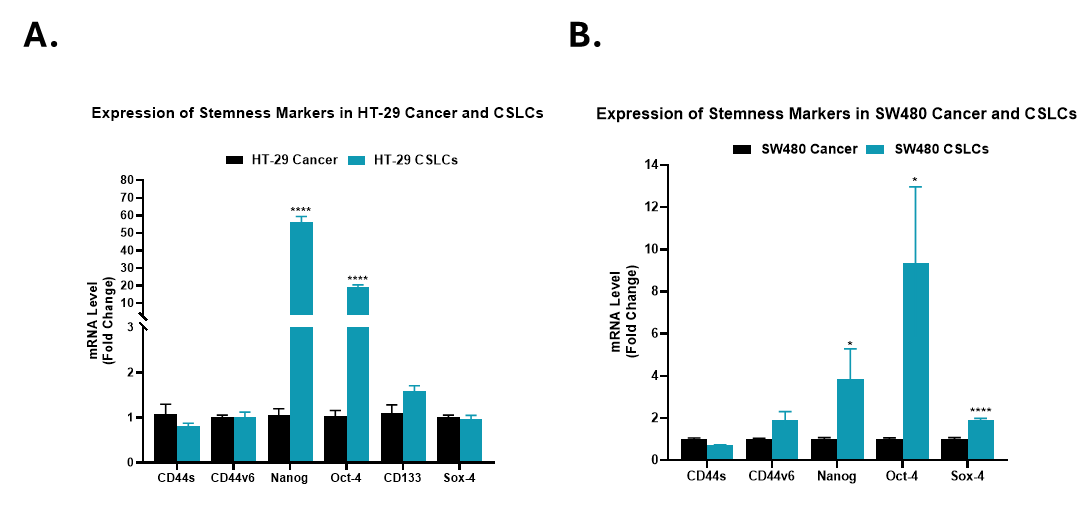


Figure S1. Expression of stemness-associated markers by RT-qPCR in CSC-enriched spheroids versus bulk cancer cells. RT-qPCR analysis of stemness-associated genes across different passages of HT-29 (A) and SW480 (B) spheroid cultures (CSLCs) compared with their respective parental adherent (cancer) cells. mRNA levels were normalized to the housekeeping gene GAPDH and are shown as fold change relative to cancer cells. Data represent mean ± SEM. **p* < 0.05, ***p* < 0.01, and ****p* < 0.001 by Student’s t-test; n ≥ 3.


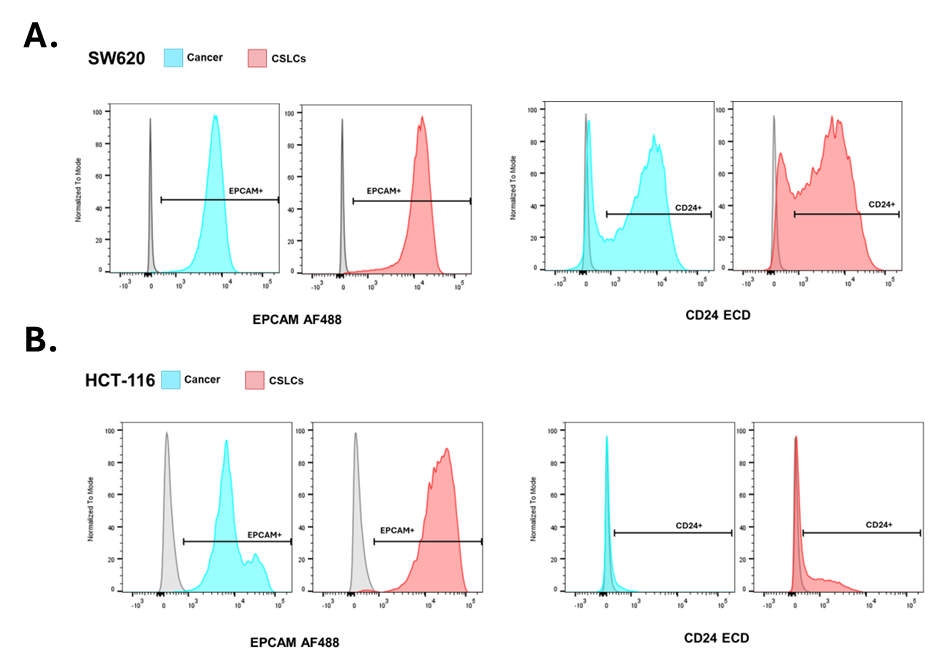


Figure S2. Surface expression of stemness-associated markers in spheroid-enriched CSLCs. Representative flow-cytometry histograms of selected stemness markers in spheroid-enriched cancer stem–like cells (CSLCs; red) compared with the corresponding adherent/parental cancer cells (blue) in (A) HCT-116 and (B) SW620. Gray histograms indicate the matched isotype control (for directly conjugated antibodies) or secondary-antibody–only control (for unconjugated primary antibodies).


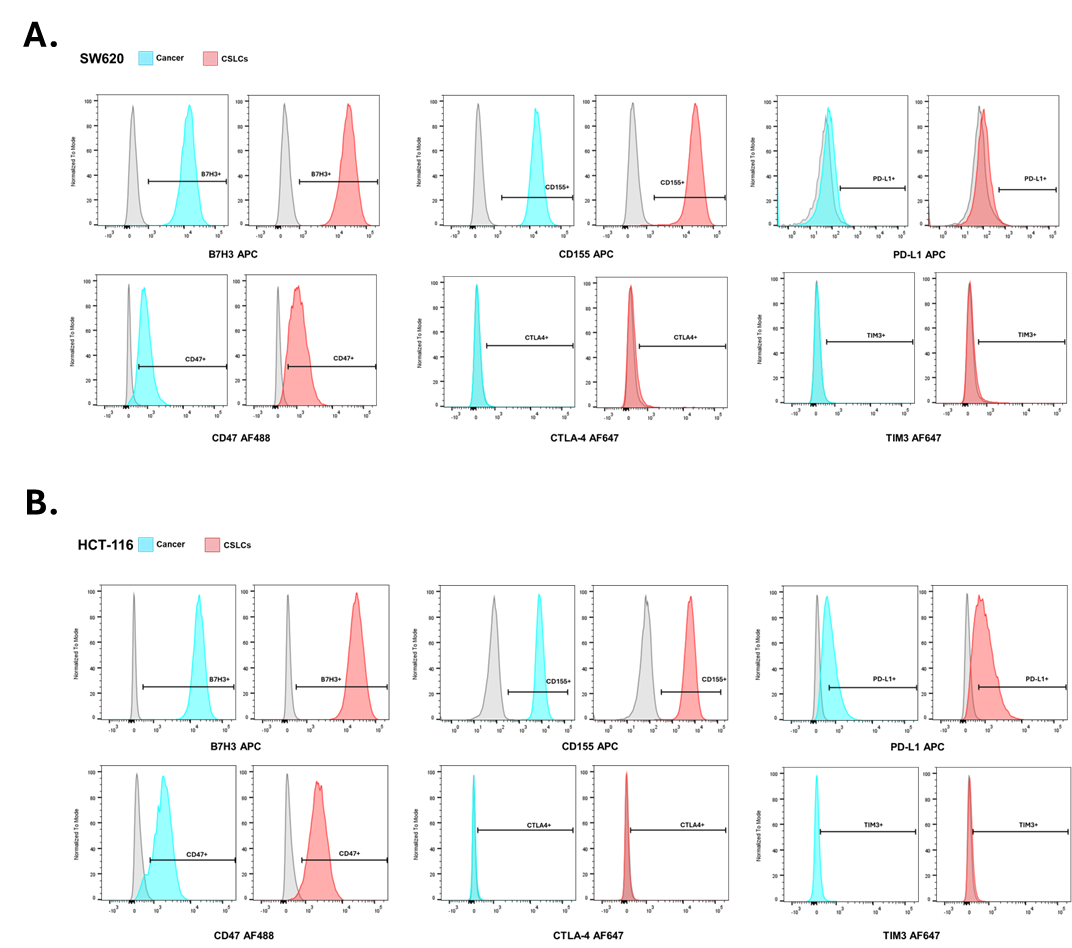


Figure S3. Surface expression of selected immune checkpoint (ICPs) in spheroid-enriched CSLCs. Representative flow-cytometry histograms of selected ICPs in spheroid-enriched cancer stem–like cells (CSLCs; red) compared with the corresponding adherent/parental cancer cells (blue) in (A) HCT-116 and (B) SW620. Gray histograms indicate the matched isotype control (for directly conjugated antibodies) or secondary-antibody–only control (for unconjugated primary antibodies).


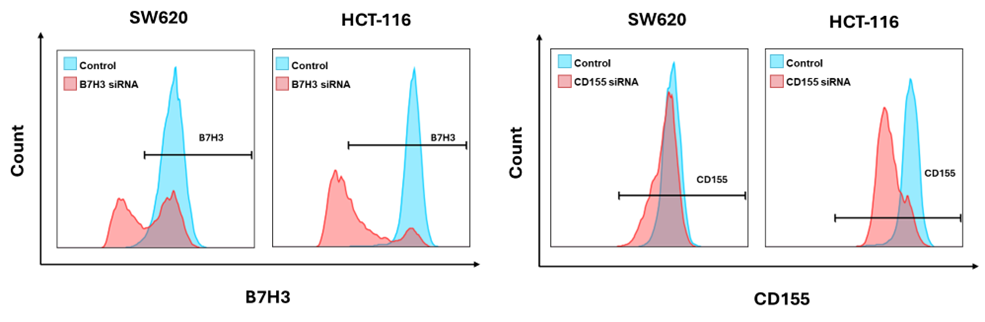


Figure S4. Validation of siRNA-mediated knockdown of B7-H3 (A) and CD155 (B) by flow cytometry. Overlaid histograms show surface expression in siRNA control cells (blue) versus cells transfected with the indicated siRNA (red).


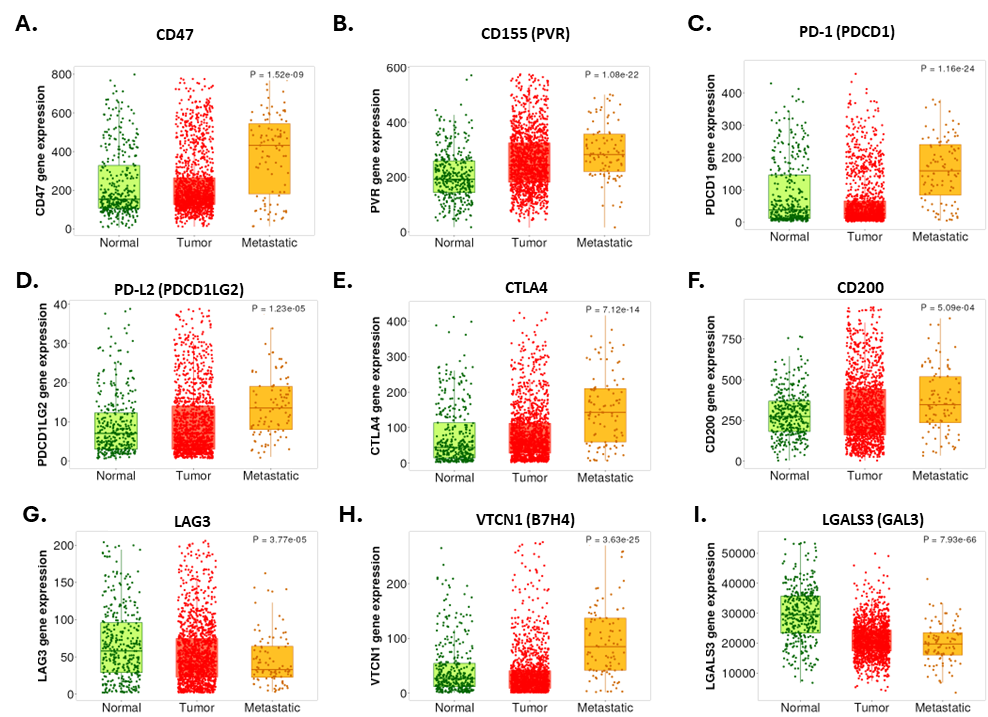


Figure S5. Box plots showing normal vs. Tumor vs. metastatic colon tissue expression of by Gene chip data, generated with TNM_plot web tool. P values (Kruskal-Wallis P) are shown.


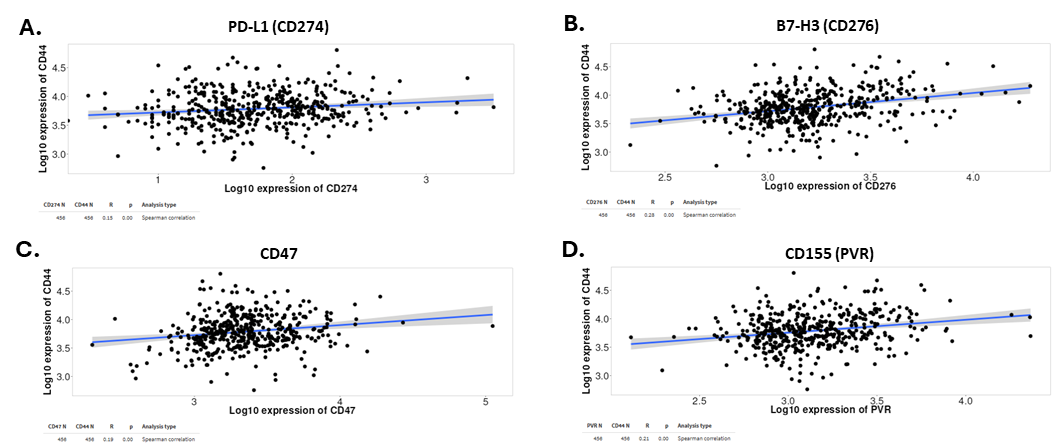


Figure S6. (A-D) Spearman correlation between CD44 expression (y-axis) and selected immune checkpoints (x-axis) in colorectal cancer patients generated using TNM_plot (https://tnmplot.com/analysis/, accessed on September 5, 2025).


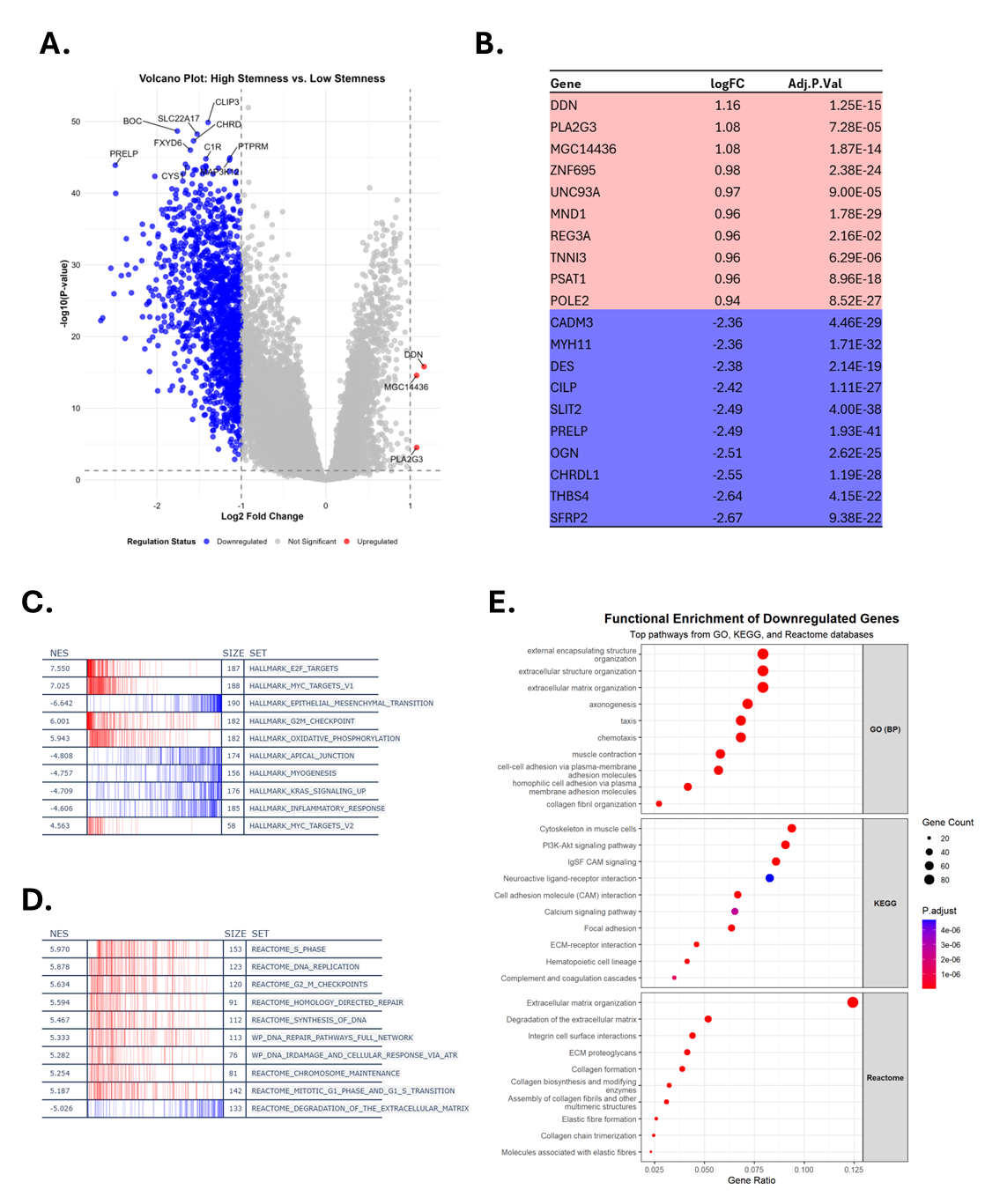


Figure S7. Identification of differentially expressed genes (DEGs) and their functional characterization across high- and low- stemness groups. (A) Volcano plot showing DEGs between high-stemness and low-stemness groups in the TCGA colorectal cancer cohort, classified based on the median mRNAsi score. Genes upregulated in high-stemness group are shown in red, significantly downregulated genes in blue, and non-significant genes in grey, using |log2FC| > 1 and p < 0.05 as cut off criteria. Top-ten highly dysregulated genes are labeled. (B) Top DEGs ranked by log2 fold change and statistical significance, highlighting the most significantly upregulated and downregulated genes in high-stemness tumors. (C, D) Gene Set Enrichment Analysis (GSEA) of Hallmark and Reactome pathways showing pathways positively enriched in high-stemness tumors (positive NES) and pathways enriched in low-stemness tumors (negative NES). (E) Functional enrichment analysis of genes downregulated in high-stemness tumors using GO Biological Process, KEGG, and Reactome databases. Dot size corresponds to gene count and color reflects adjusted p-value.


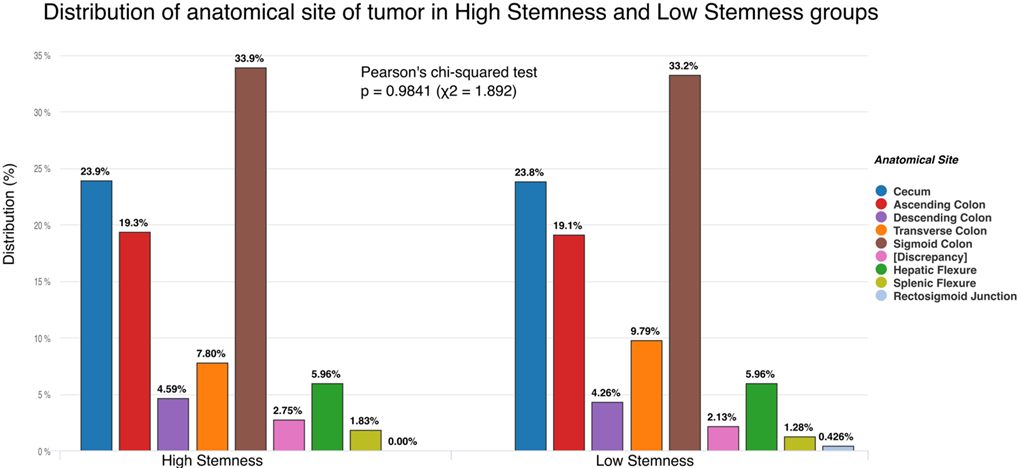


Figure S8 Distribution of anatomical site of tumor in high stemness and low stemness groups..


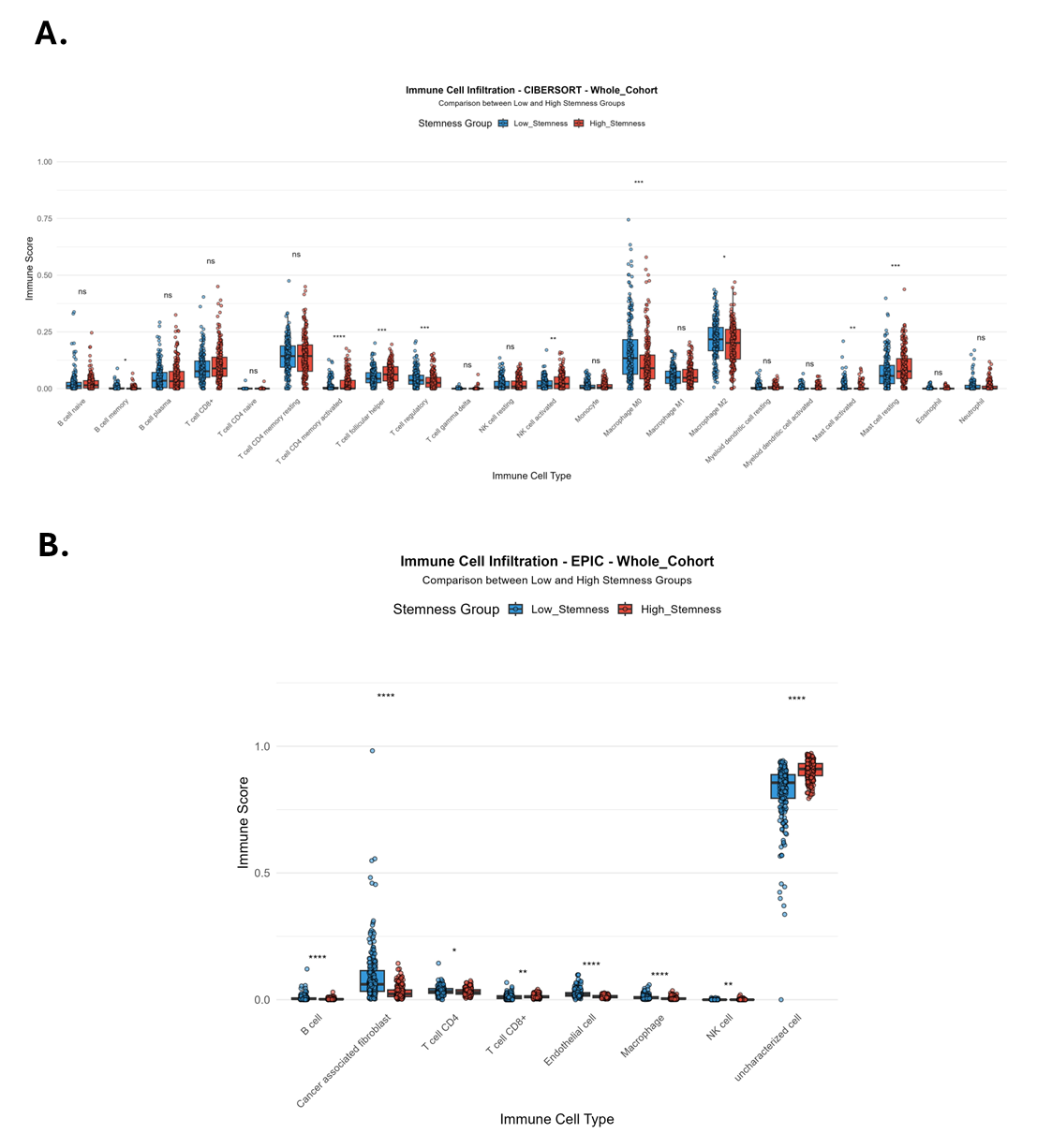


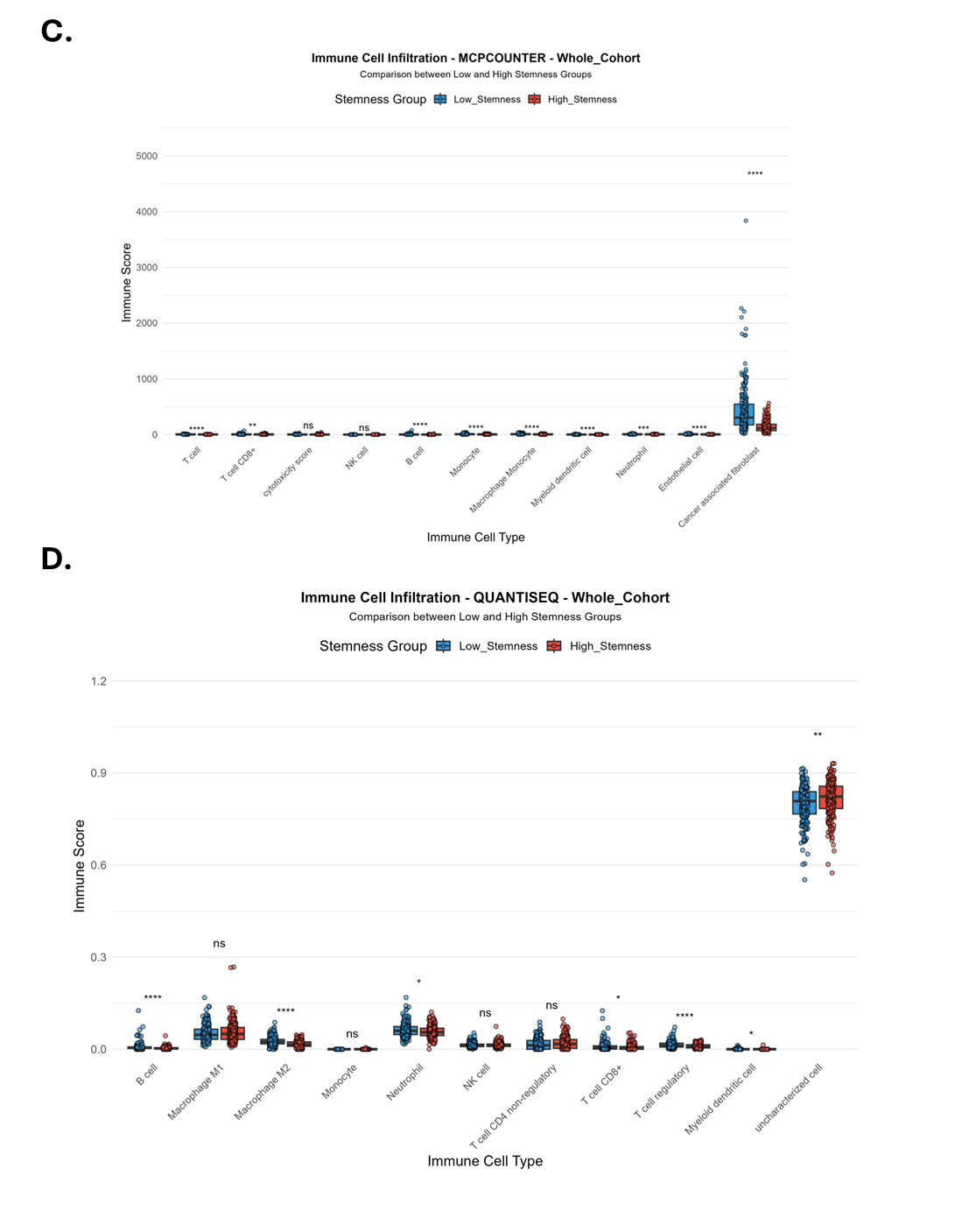


**E.**


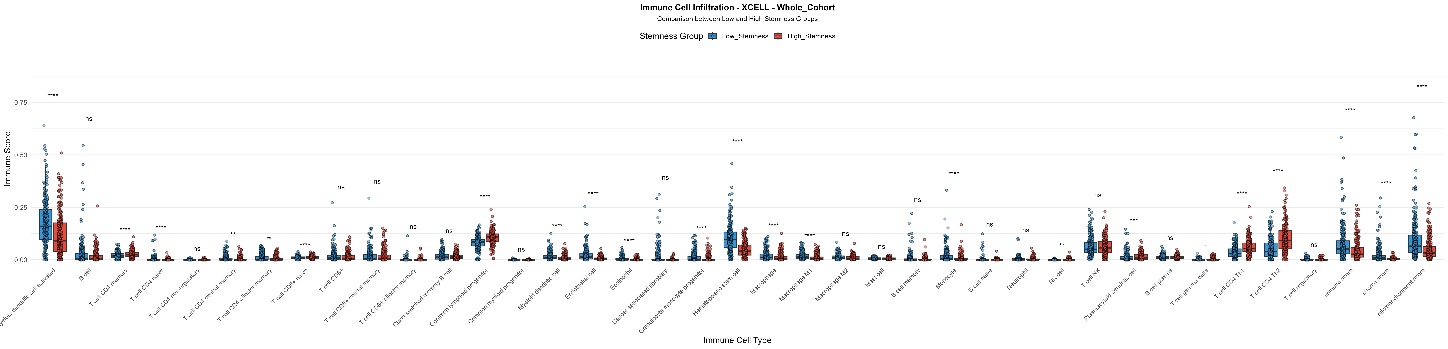


Figure S9. Comparison of tumor-infiltrating lymphocytes (TILs) between low- and high-stemness groups based on the CIBERSORT (A), EPIC (B), MCPCOUNTER (C), QUANTISEQ (D), XCELL (E) algorithms.


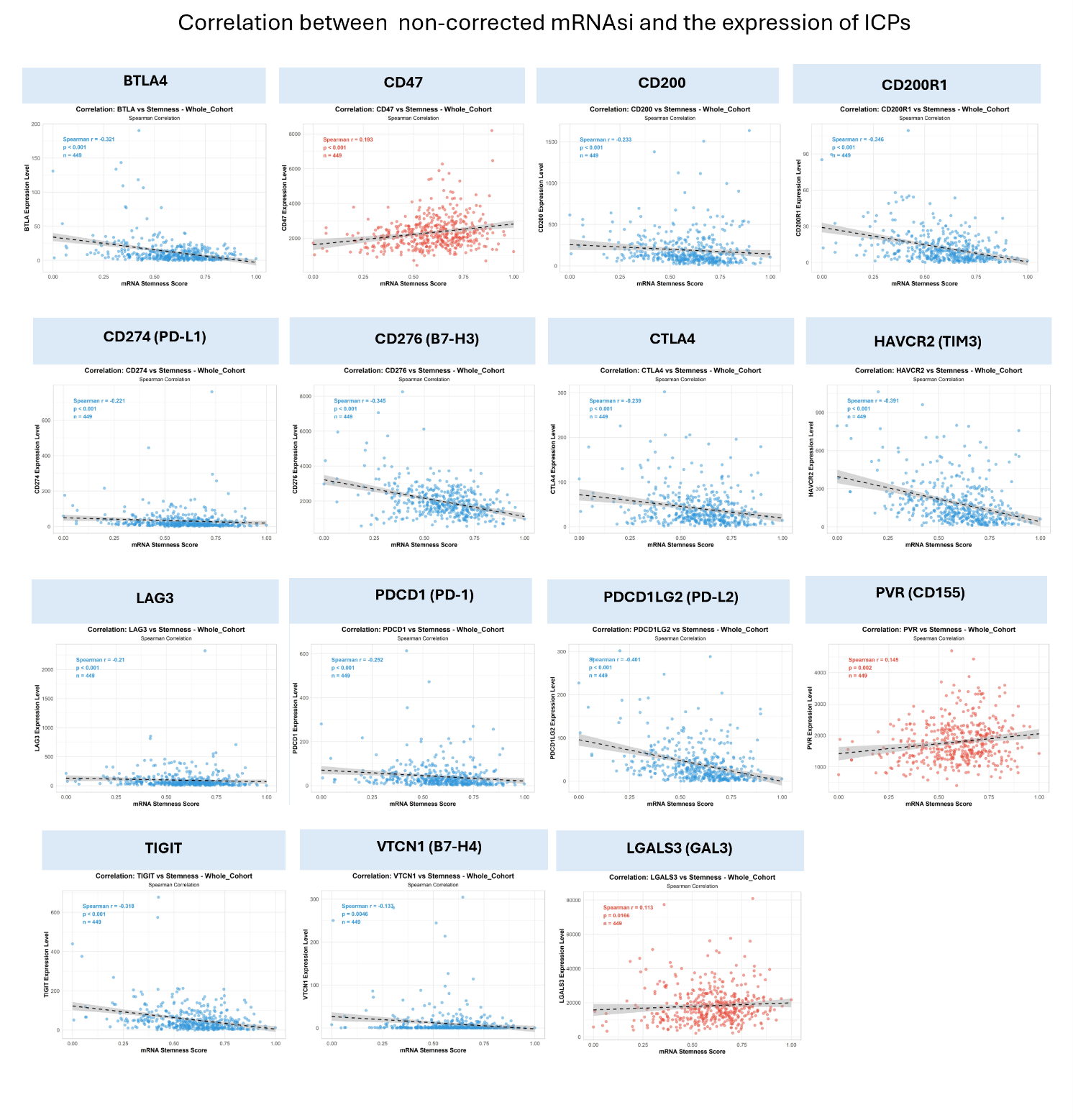


Figure S10. Correlation between non-corrected mRNAsi and the expression of selected inhibitory immune checkpoints (ICPs)


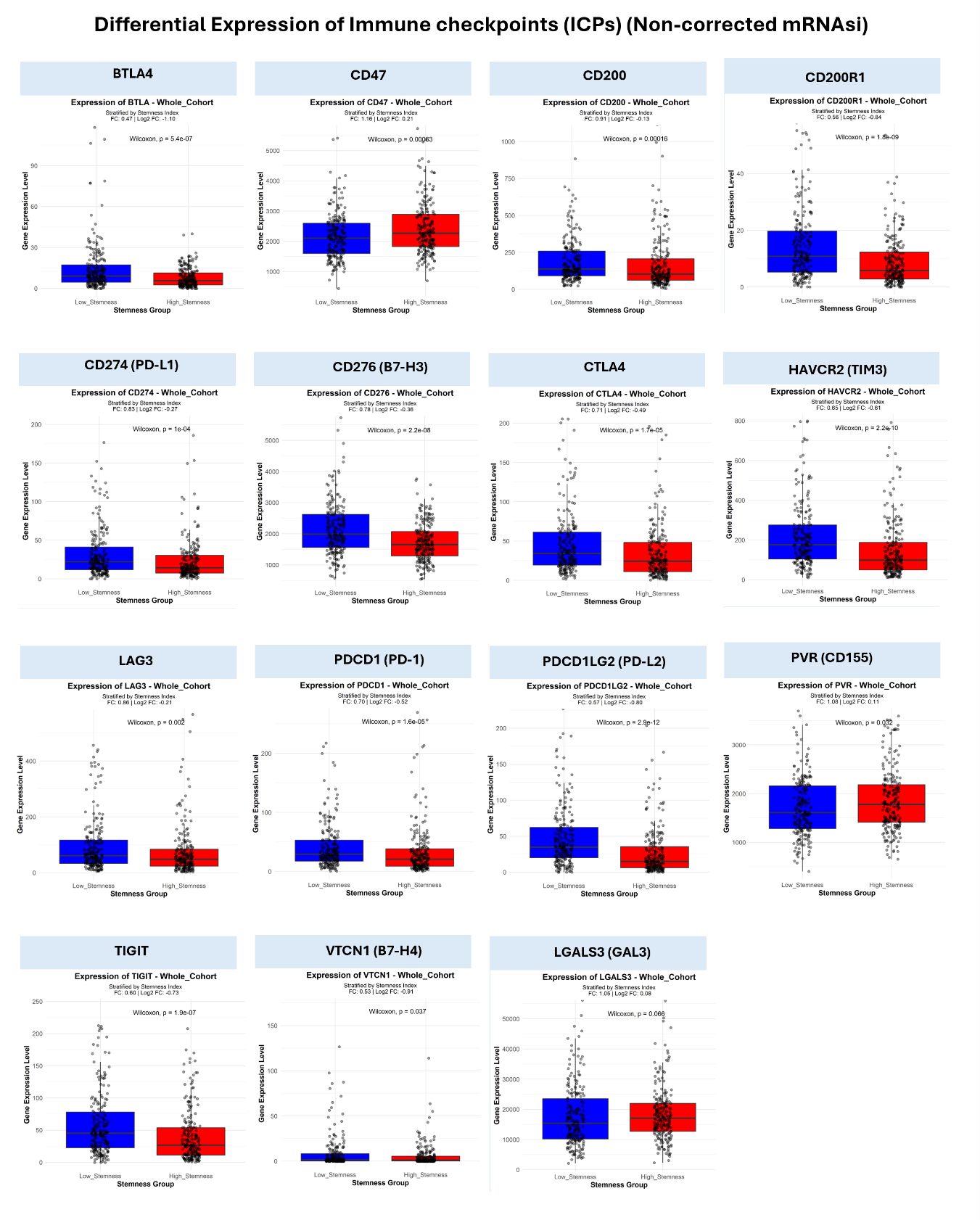


Figure S11. Differential expression of Immune checkpoints (ICPs) between low stemness and high stemness groups stratified based on non-corrected mRNAsi score.


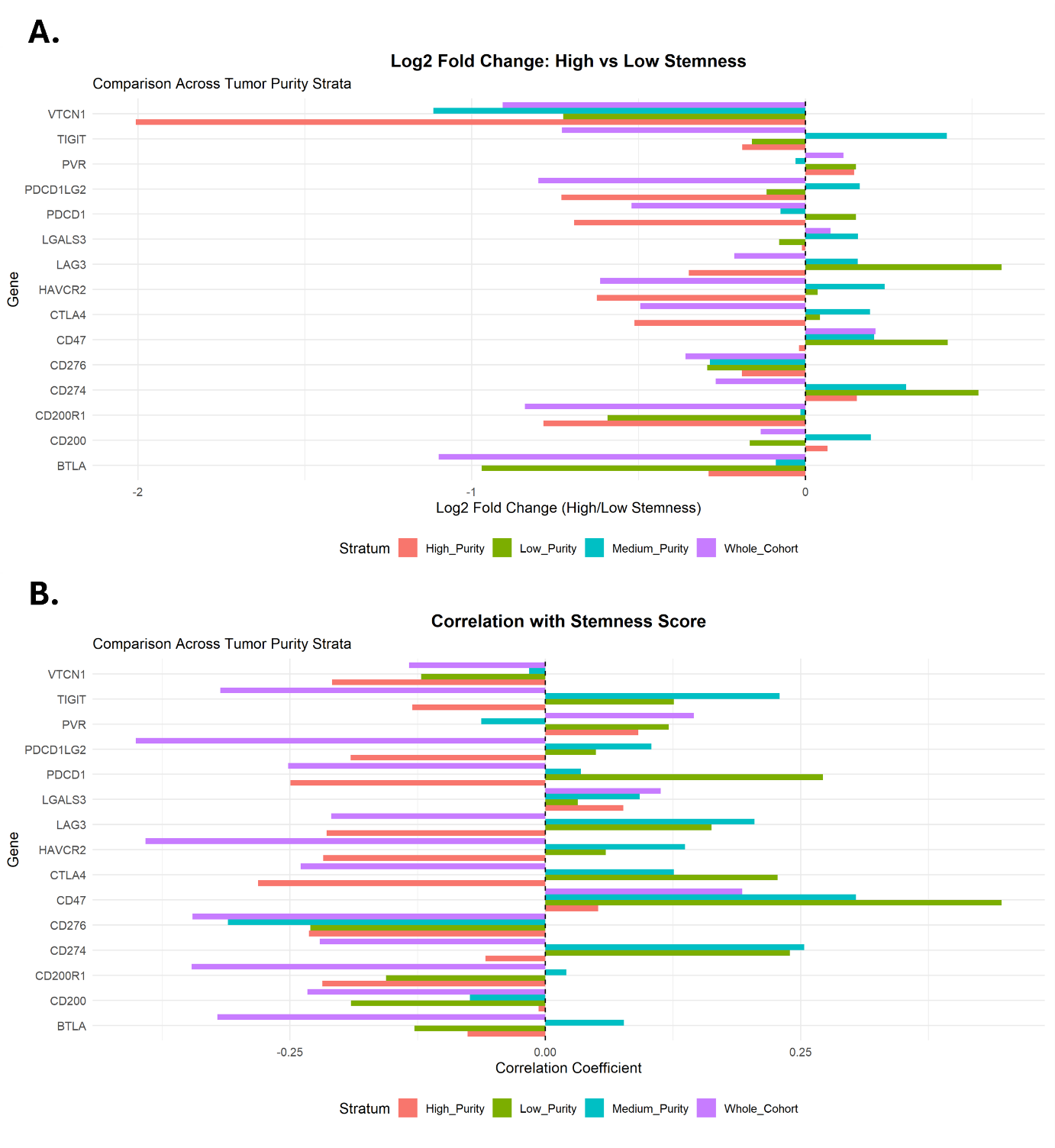


Figure S12. (A) Log2 fold-change of inhibitory immune checkpoint genes between high- and low-stemness tumors across tumor-purity strata. Horizontal bar plots show the log2 fold-change (high-stemness vs. low-stemness) for each immune checkpoint gene within the TCGA CRC cohort, stratified by tumor purity (high-purity, medium-purity, and low-purity groups), alongside the whole-cohort analysis. (B) Correlation between inhibitory immune checkpoint expression and stemness score across tumor-purity strata. Bar plots display the correlation coefficients between each immune checkpoint gene and the mRNAsi stemness score within the TCGA CRC cohort. Analyses were stratified by tumor purity (high-, medium-, and low-purity groups), with the whole-cohort correlation shown for comparison.


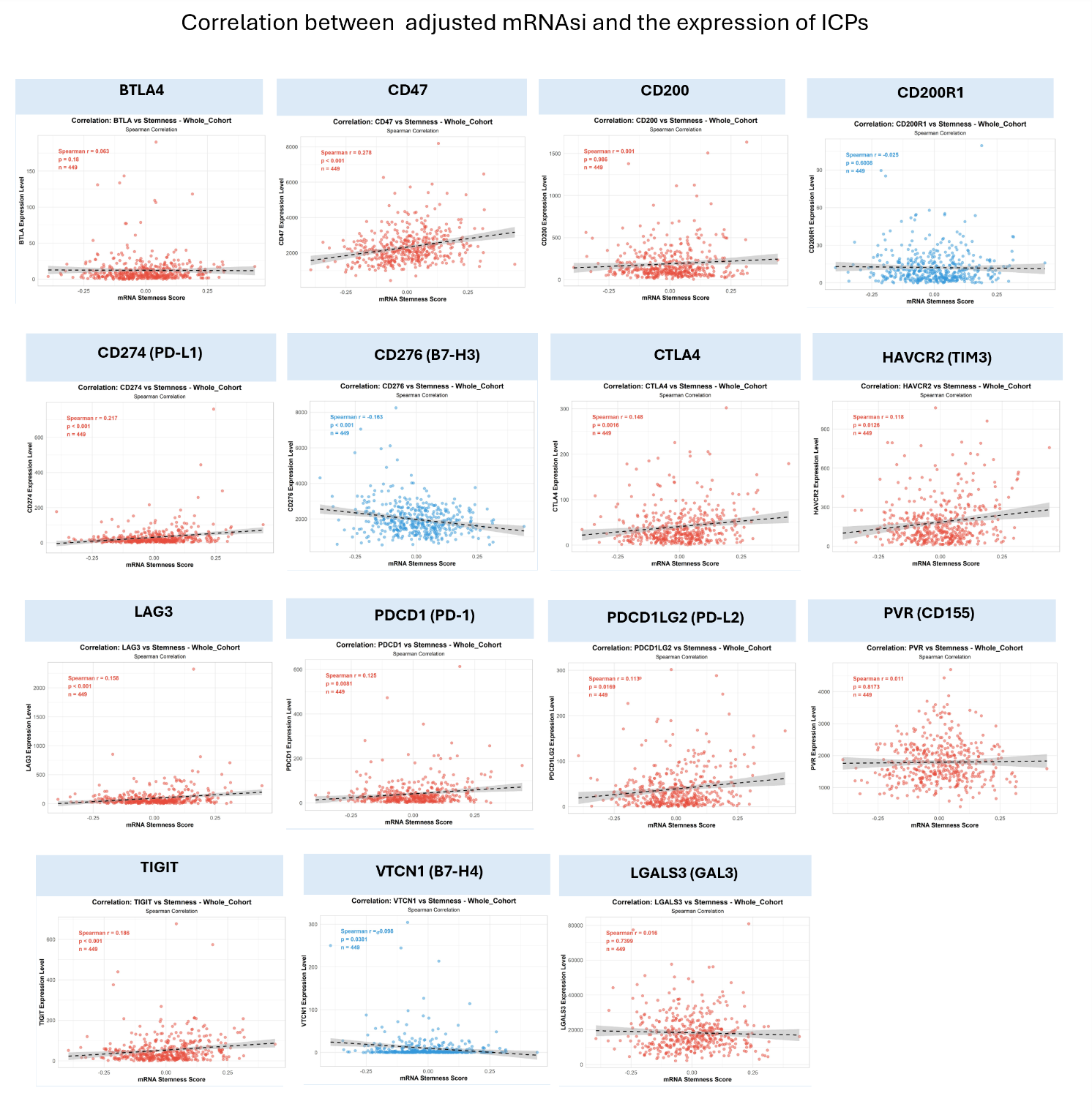


Figure S13. Correlation between tumor-purity adjusted mRNAsi and the expression of selected inhibitory immune checkpoints (ICPs).


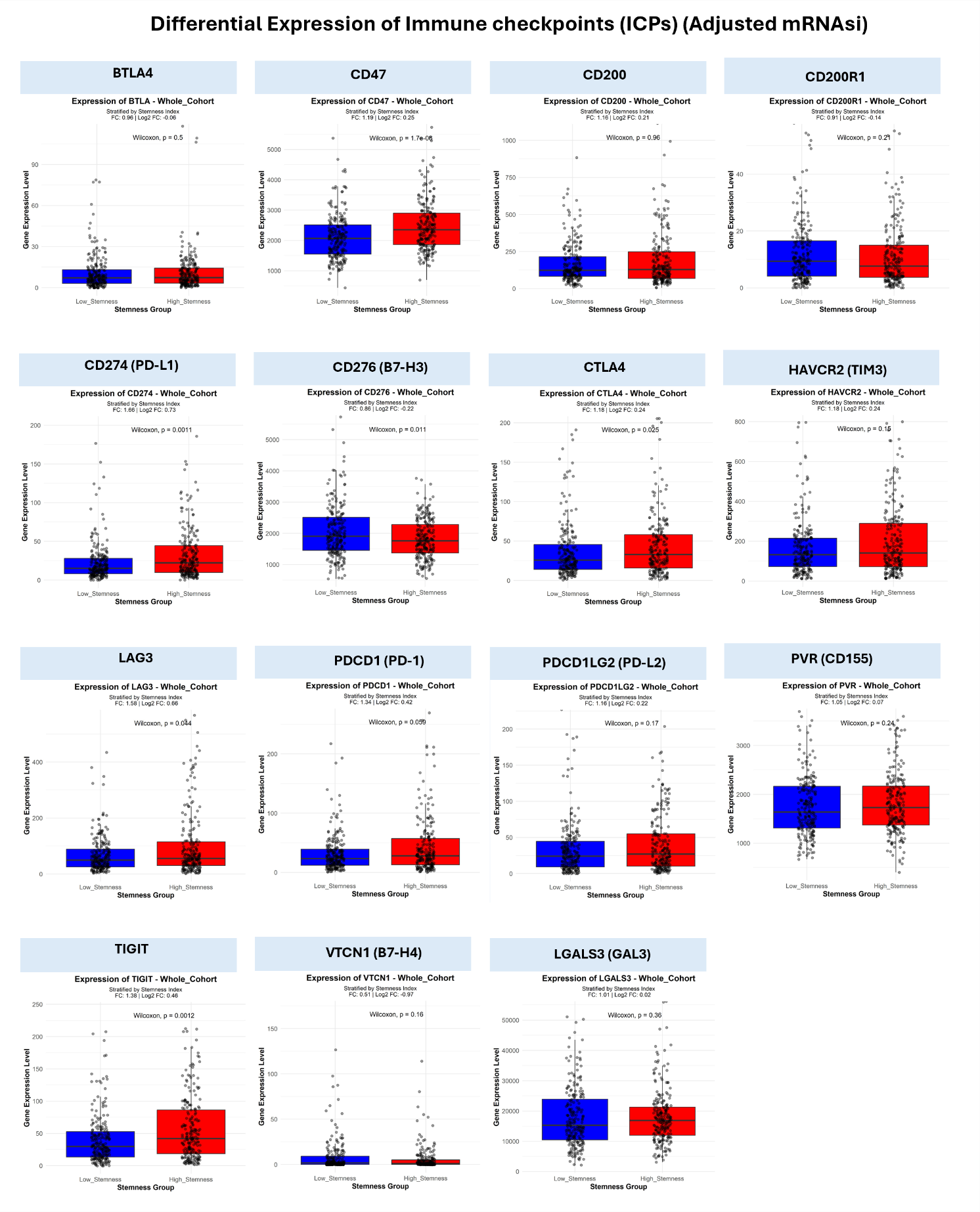


Figure S14. Differential expression of Immune checkpoints (ICPs) between low stemness and high stemness groups stratified based on tumor-purity adjusted mRNAsi score.
